# Supplementary material for: Differential attainment in specialty training recruitment in the UK: an observational analysis of the impact of psychometric testing assessment in Public Health postgraduate selection
Source: BMJ Open. 2023 Mar 9;13(3):e069738. doi: 10.1136/bmjopen-2022-069738 (PMC10008157; doi:10.1136/bmjopen-2022-069738)
Supplement: Supplementary data [file bmjopen-2022-069738supp001.pdf]

Differential attainment in specialty training recruitment in the United Kingdom: an observational analysis of the impact of psychometric testing assessment in Public Health postgraduate selection.

Submission for BMJ OPEN

STRICTLY CONFIDENTIAL

Authors

Richard J Pinder[1] **Corresponding author; richard.pinder@imperial.ac.uk**

ORCID: 0000-0002-7010-6009;

Fran Bury[1] ORCID: 0000-0002-6305-1994

Ganesh Sathyamoorthy[1,2, 3]

Azeem Majeed[1,3] ORCID: 0000-0002-2357-9858

Mala Rao[1,2,3] ORCID: 0000-0001-5504-6303

Affiliations

1. Department of Primary Care and Public Health, School of Public Health, Imperial College London
2. Ethnicity and Health Unit, Department of Primary Care and Public Health, Imperial College London
3. NIHR Applied Research Collaboration Northwest London

Key words

Public health, recruitment, medical training, ethnicity, differential attainment

SUPPLEMENTARY MATERIALS

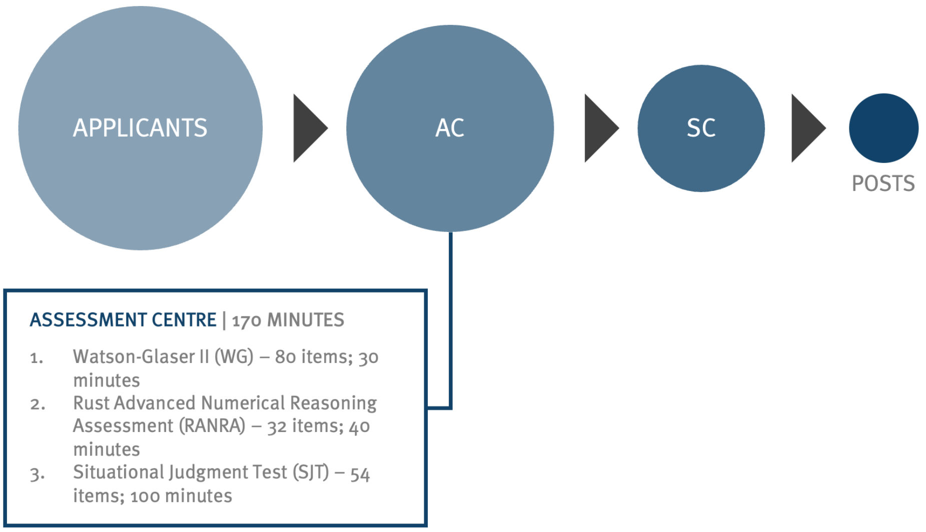

Supplementary Figure 1. Recruitment cycle diagram for Public Health specialty training, 2009-2020

Pinder RJ, *et al. BMJ Open* 2023; 13:e069738. doi: 10.1136/bmjopen-2022-069738

Supplementary Table 1. Univariable and multivariable logistic regression of demographic associations of candidates passing RANRA (count, percentage, odds ratios, adjusted odds ratios, 95% confidence intervals and p-values).

	Passed RANRA (% of those sitting)	Odds ratio (95% CI)	p-value	Adjusted odds ratio* (95% CI)	p-value
**Overall**	499 (79.3)				
**Sex**					
- Male	157 (78.9)	Ref		Ref	
- Female	324 (78.8)	1.00 (0.66 to 1.51)	0.99	1.06 (0.59 to 1.90)	0.85
**Ethnic Group**					
- White British	270 (88.8)	Ref		Ref	
- White Other	47 (79.7)	0.49 (0.24 to 1.02)	0.06	0.90 (0.33 to 2.48)	0.85
- Black	31 (55.4)	0.16 (0.08 to 0.30)	<0.001	0.19 (0.08 to 0.44)	<0.001
- Asian	79 (69.3)	0.28 (0.17 to 0.49)	<0.001	0.35 (0.16 to 0.71)	<0.001
- Mixed	25 (78.1)	0.45 (0.18 to 1.11)	0.09	0.63 (0.20 to 1.99)	0.43
- Chinese	8 (72.7)	0.34 (0.08 to 1.33)	0.12	0.55 (0.05 to 6.21)	0.63
- Other	10 (66.7)	0.25 (0.08 to 0.78)	0.02	0.31 (0.07 to 1.36)	0.12
**Age in years****	Range: 24 to 55	0.92 (0.89 to 0.95)	<0.001	0.95 (0.91 to 0.99)	0.02
**Parent or carer highest qualification**					
- No degree	180 (79.3)	Ref		Ref	
- Degree	300 (79.6)	1.02 (0.68 to 1.53)	0.93	1.16 (0.67 to 1.77)	0.60
**Main language**					
- English	363 (80.9)	Ref		Ref	
- Not English	19 (61.3)	0.38 (0.18 to 0.80)	0.01	0.66 (0.25 to 1.77)	0.41
**Professional background**					
- BOTM	251 (74.5)	0.14 (0.07 to 0.28)	<0.001	0.18 (0.07 to 0.47)	<0.001
- IMG	39 (53.4)	0.05 (0.03 to 0.12)	<0.001	0.10 (0.04 to 0.32)	<0.001
- UK Medical	209 (95.4)	Ref		Ref	

* - adjustment includes sex, ethnic group, age, parent or carer degree status, main language and professional background.
** - age as a continuous variable in years.

Pinder RJ, *et al. BMJ Open* 2023; 13:e069738. doi: 10.1136/bmjopen-2022-069738

Supplementary Table 2. Univariable and multivariable logistic regression of demographic associations of candidates passing Watson-Glaser (count, percentage, odds ratios, adjusted odds ratios, 95% confidence intervals and p-values).

	Passed WG (% of those sitting)	Odds ratio (95% CI)	p-value	Adjusted odds ratio* (95% CI)	p-value
**Overall**	466 (74.1)				
**Sex**					
- Male	140 (70.4)	Ref			
- Female	310 (75.4)	1.29 (0.89-1.89)	0.18	1.25 (0.73-2.17)	0.41
**Ethnic Group**					
- White British	255 (83.9)	Ref		Ref	
- White Other	47 (79.7)	0.75 (0.37-1.52)	0.43	1.22 (0.45-3.30)	0.70
- Black	26 (46.4)	0.17 (0.91-0.31)	<0.001	0.20 (0.09-0.46)	<0.001
- Asian	72 (63.2)	0.33 (0.20-0.54)	<0.001	0.30 (0.16-0.58)	<0.001
- Mixed	26 (81.3)	0.83 (0.33-2.13)	0.70	1.24 (0.36-4.30)	0.73
- Chinese	6 (54.6)	0.23 (0.68-0.79)	0.02	0.22 (0.03-1.74)	0.15
- Other	8 (53.3)	0.22 (0.08-0.63)	0.01	0.25 (0.06-1.00)	0.05
**Age in years****	Range: 24-54	0.94 (0.92-0.97)	<0.001	0.95 (0.92-1.00)	0.03
**Parent or carer highest qualification**					
- No degree	159 (70.0)	Ref		Ref	
- Degree	287 (76.1)	1.36 (0.94-1.97)	0.10	1.78 (1.07-2.98)	0.03
**Main language**					
- English	347 (77.3)	Ref		Ref	
- Not English	19 (61.3)	0.47 (0.22-0.99)	0.05	0.84 (0.31-2.23)	0.72
**Professional background**					
- BOTM	243 (72.1)	0.35 (0.22-0.56)	<0.001	0.39 (0.19-0.80)	0.01
- IMG	30 (41.1)	0.09 (0.05-0.17)	<0.001	0.13 (0.05-0.33)	<0.001
- UK Medical	193 (88.1)	Ref		Ref	

* - adjustment includes sex, ethnic group, age, parent or carer degree status, main language and professional background.
** - age as a continuous variable in years.

Supplementary Table 3. Univariable and multivariable logistic regression of demographic associations of candidates passing Situational Judgment Test (count, percentage, odds ratios, adjusted odds ratios, 95% confidence intervals and p-values).

	Passed SJT (% of those sitting)	Odds ratio (95% CI)	p-value	Adjusted odds ratio* (95% CI)	p-value
**Overall**	465 (73.9)				
**Sex**					
- Male	143 (71.9)	Ref		Ref	
- Female	307 (74.7)	1.16 (0.79-1.69)	0.46	1.59 (0.94-2.69)	0.09
**Ethnic Group**					
- White British	251 (82.6)	Ref		Ref	
- White Other	44 (74.6)	0.62 (0.32-1.19)	0.15	1.25 (0.49-3.21)	0.64
- Black	30 (53.6)	0.24 (0.13-0.45)	<0.001	0.39 (0.17-0.88)	0.02
- Asian	73 (64.0)	0.38 (0.23-0.61)	<0.001	0.38 (0.20-0.74)	0.01
- Mixed	22 (68.8)	0.46 (0.21-1.04)	0.06	0.74 (0.26-2.11)	0.57
- Chinese	7 (63.6)	0.37 (0.10-1.31)	0.12	0.26 (0.03-2.24)	0.22
- Other	9 (60.0)	0.32 (0.11-0.93)	0.04	0.30 (0.07-1.22)	0.09
**Age in years****	Range: 24-50	0.93 (0.90-0.95)		0.97 (0.93-1.01)	0.11
**Parent or carer highest qualification**					
- No degree	164 (72.3)	Ref		Ref	
- Degree	280 (74.3)	1.11 (0.77-1.61)	0.59	1.36 (0.83-2.23)	0.22
**First language**					
- English	339 (75.5)	Ref		Ref	
- Other	17 (54.8)	0.39 (0.19-0.83)	0.01	1.02 (0.68-2.71)	0.97
**Professional background**					
- BOTM	227 (67.4)	0.15 (0.09-0.27)	<0.001	0.16 (0.07-0.35)	<0.001
- IMG	34 (46.6)	0.06 (0.03-0.13)	<0.001	0.07 (0.03-0.19)	<0.001
- UK Medical	204 (93.2)	Ref		Ref	

* - adjustment includes sex, ethnic group, age, parent or carer degree status, main language and professional background.
** - age as a continuous variable in years.
