# Supplementary material for: Differential attainment in specialty training recruitment in the UK: an observational analysis of the impact of psychometric testing assessment in Public Health postgraduate selection
Source: BMJ Open. 2023 Mar 9;13(3):e069738. doi: 10.1136/bmjopen-2022-069738 (PMC10008157; doi:10.1136/bmjopen-2022-069738)
Supplement: Author's manuscript [file bmjopen-2022-069738.draft_revisions.pdf]

BMJ Open is committed to open peer review. As part of this commitment we make the peer review history of every article we publish publicly available.

When an article is published we post the peer reviewers' comments and the authors' responses online. We also post the versions of the paper that were used during peer review. These are the versions that the peer review comments apply to.

The versions of the paper that follow are the versions that were submitted during the peer review process. They are not the versions of record or the final published versions. They should not be cited or distributed as the published version of this manuscript.

BMJ Open is an open access journal and the full, final, typeset and author-corrected version of record of the manuscript is available on our site with no access controls, subscription charges or pay-per-view fees (http://bmjopen.bmj.com).

If you have any questions on BMJ Open's open peer review process please email info.bmjopen@bmj.com

BMJ Open

Differential attainment in specialty training recruitment in the United Kingdom: an observational analysis of the impact of psychometric testing assessment in Public Health postgraduate selection.

Journal:	*BMJ Open*
Manuscript ID	bmjopen-2022-069738
Article Type:	Original research
Date Submitted by the Author:	22-Nov-2022
Complete List of Authors:	Pinder, Richard; Imperial College London School of Public Health, Department of Primary Care and Public Health<br>Bury, Fran; Imperial College London<br>Sathyamoorthy, Ganesh; Imperial College London, NIHR Northwest London Applied Research Collaboration<br>Majeed, Azeem; Imperial College London, Primary Care<br>Rao, Mala; Imperial College London, Department of Public Health
Keywords:	Human resource management < HEALTH SERVICES ADMINISTRATION & MANAGEMENT, PUBLIC HEALTH, Health policy < HEALTH SERVICES ADMINISTRATION & MANAGEMENT

SCHOLARONE™
Manuscripts

I, the Submitting Author has the right to grant and does grant on behalf of all authors of the Work (as defined in the below author licence), an exclusive licence and/or a non-exclusive licence for contributions from authors who are: i) UK Crown employees; ii) where BMJ has agreed a CC-BY licence shall apply, and/or iii) in accordance with the terms applicable for US Federal Government officers or employees acting as part of their official duties; on a worldwide, perpetual, irrevocable, royalty-free basis to BMJ Publishing Group Ltd ("BMJ") its licensees and where the relevant Journal is co-owned by BMJ to the co-owners of the Journal, to publish the Work in this journal and any other BMJ products and to exploit all rights, as set out in our licence.

The Submitting Author accepts and understands that any supply made under these terms is made by BMJ to the Submitting Author unless you are acting as an employee on behalf of your employer or a postgraduate student of an affiliated institution which is paying any applicable article publishing charge ("APC") for Open Access articles. Where the Submitting Author wishes to make the Work available on an Open Access basis (and intends to pay the relevant APC), the terms of reuse of such Open Access shall be governed by a Creative Commons licence – details of these licences and which Creative Commons licence will apply to this Work are set out in our licence referred to above.

Other than as permitted in any relevant BMJ Author's Self Archiving Policies, I confirm this Work has not been accepted for publication elsewhere, is not being considered for publication elsewhere and does not duplicate material already published. I confirm all authors consent to publication of this Work and authorise the granting of this licence.

For peer review only

Differential attainment in specialty training recruitment in the United Kingdom: an observational analysis of the impact of psychometric testing assessment in Public Health postgraduate selection.

Submission for BMJ OPEN

STRICTLY CONFIDENTIAL

Authors

Richard J Pinder[1] **Corresponding author; richard.pinder@imperial.ac.uk**

ORCID: 0000-0002-7010-6009;

Fran Bury[1] ORCID: 0000-0002-6305-1994

Ganesh Sathyamoorthy[1,2, 3]

Azeem Majeed[1,3] ORCID: 0000-0002-2357-9858

Mala Rao[1,2,3] ORCID: 0000-0001-5504-6303

Affiliations

1. Department of Primary Care and Public Health, School of Public Health, Imperial College London
2. Ethnicity and Health Unit, Department of Primary Care and Public Health, Imperial College London
3. NIHR Applied Research Collaboration Northwest London

Key words

Public health, recruitment, medical training, ethnicity, differential attainment

3177 words excluding abstract, 1 box and 3 tables.
Supplementary data: 1 figure and 3 tables.

For peer review only - http://bmjopen.bmj.com/site/about/guidelines.xhtml
For peer review only

ABSTRACT (297 words)

Objectives

To determine how current psychometric testing approaches used in selection to postgraduate training in UK Public Health is associated with socioeconomic and sociocultural background of applicants (including ethnicity).

Design

Observational study using contemporaneous data collected during recruitment and psychometric test scores.

Setting

Assessment Centre of UK national Public Health recruitment for postgraduate Public Health training. The Assessment Centre element of selection comprises three psychometric assessments: Rust Advanced Numerical Reasoning (RANRA), Watson Glaser Critical Thinking Assessment II (WGCTAII) and Public Health situational judgment test (SJT).

Participants

629 applicants completed the Assessment Centre in 2021. 219 (34.8%) were UK medical graduates, 73 (11.46%) were international medical graduates, and 337 (53.6%) were from backgrounds other than medicine.

Main outcome measure

Multivariable adjusted progression statistics in the form of adjusted odds ratio (aOR), accounting for age, sex, ethnicity, professional background and surrogate measures of familial socioeconomic and sociocultural status.

Results

357 (56.8%) candidates scored passed all three psychometric tests to progress. Candidate characteristics associated with lower progression were Black ethnicity (aOR 0.19, 0.08 to 0.44), Asian ethnicity (aOR 0.35, 0.16-0.71) and coming from a non-UK medical graduate background (aOR 0.05, 0.03 to 0.12); similar differential attainment was observed in each of the psychometric tests. Even within the UK-trained medical cohort, candidates from White British backgrounds were more likely to progress than those from ethnic minorities (89.2% versus 75.0%, p=0.003).

Conclusion

For peer review only - http://bmjopen.bmj.com/site/about/guidelines.xhtml

For peer review only

Although perceived to mitigate the risks of conscious and unconscious bias in selection to medical postgraduate training, psychometric tests demonstrate unwarranted and unacceptable differential attainment. While other specialities should enhance their data collection to evaluate the impact of differential attainment on current selection processes, the roll-out of the psychometric Multi Specialty Recruitment Assessment (MSRA) across further specialities in the UK should be urgently explored in respect of differential attainment.

STRENGTHS AND LIMITATIONS OF THIS STUDY

- This study analyses a complete annual cohort of applicants for UK Public Health specialty training in 2021 to identify the extent and impact of differential attainment arising in the psychometric testing component of selection.
- The methodology employs an enhanced and prospectively-collected set of questions that explore sociocultural background of applicants, as well as categorising UK medical graduates, international medical graduates and candidates from backgrounds other than medicine.
- Socioeconomic disadvantage and intersectionality are inherently complex and difficult to quantify; we cannot exclude the possibility of other potential confounders.

For peer review only - http://bmjopen.bmj.com/site/about/guidelines.xhtml

INTRODUCTION (739 words)

Over recent years, awareness of the challenges facing doctors and other healthcare workers from ethnic minority backgrounds has increased.[1] The inequity of opportunity and outcome is driven by myriad factors: through barriers to professional progression;[2–4] more active forms of racism from patients, colleagues and institutions;[5] as well as through the interaction with other social categorisations (so-called intersectionality).[6] Differential attainment describes a gap in attainment levels between different demographic groups, including by protected characteristics.[7] Differential attainment is present in medical school selection,[8] during undergraduate medical training,[9] in postgraduate training[10] in senior appointments,[11] and in the field of medical research.[12]

Like other employment sectors, the medical profession internationally has tried to mitigate unconscious bias arising during recruitment.[13] As part of the historical shift from resource-intensive interviews where unconscious (and potentially even conscious) bias has been suspected,[14] psychometric testing has been commonly perceived as more objective.[2] And while validation of psychometric testing in the context of United Kingdom (UK) General Practice recruitment[15] and Public Health recruitment[16] has been published, conceptual validity has been largely predicated on subsequent professional progression as opposed to investigating potential differential attainment.

The use and weighting accorded to psychometric tests for postgraduate medical recruitment the UK varies. The Multi-Specialty Recruitment Assessment[17] (MSRA) is a multi-instrument psychometric assessment that began in general practice and which has also been used in ophthalmology and obstetrics and gynaecology for several years.[18] In 2022, around 10 UK specialties are using MSRA as a means of shortlisting candidates.

Public Health is an unusual specialty that recruits both medical and non-medical candidates. The latter are referred to as candidates from *backgrounds other than medicine* (BOTM). The current three-stage design of postgraduate specialty recruitment for Public Health (Supplementary Figure 1) comprises eligibility checking, an assessment centre (AC) comprising three psychometric instruments followed by a selection centre (SC); this process reduces approximately 1000 applicants to 650 applicants at AC and 216 applicants at SC.[16,19] This selection process preceded the introduction of the MSRA.

For peer review only - http://bmjopen.bmj.com/site/about/guidelines.xhtml

Due to the diversity of Public Health's applicant pool, MSRA (which assesses elements of clinical decision making) would not be a fair assessment tool. Instead, since 2009 Public Health has used two generic psychometric instruments that are used internationally in recruiting to corporate roles: Rust Advanced Numerical Reasoning Assessment[20] (RANRA) and Watson Glaser Critical Thinking Assessment II[21] (WGCTAII). A third bespoke situational judgment test (SJT) instrument is designed and implemented by Work Psychology Group with input from Public Health subject matter experts and was added to the process in 2011. The three instruments are undertaken as a 170-minute computer-based test. Much-like MSRA, the Public Health AC is operated in multiple locations by the commercial testing company PearsonVue.

In 2020, the BMJ published a news story suggesting that for every specialty included, white candidates were more likely to be deemed appointable for specialty training than ethnic minority candidates.[22] Among the specialties considered, Public Health had the greatest differential between white and ethnic minority candidates in the proportion deemed appointable. Prompted by the article, this research was independently commissioned by the UK Public Health Recruitment Executive Group (REG, a committee of Health Education England) to determine whether some groups of candidates for Public Health specialty training were less likely to be successful in their applications than others, and to determine at which stage(s) of the process this occurs. The analysis, drafting and decision to submit this paper was undertaken exclusively by the authors. This study forms the second and final paper of the commissioned work.

Our preceding paper[19] analysed 2252 applicants over the three years 2018-20 inclusive of the three-stage process. Contrary to initial beliefs that the SC interviews might be the source of the differential attainment, the greatest differential attainment was associated with psychometric stage of assessment.[19] Applicants from black backgrounds and Asian backgrounds, as well as those from BOTM were between 70% and 90% less likely to progress from the psychometric testing stage than white British candidates and medical candidates respectively. As a result of these observations, we considered residual confounding as a possible explanation, recognising our inability to disaggregate international medical graduates (who are less likely to be white British) as well as other socioeconomic differences. In this paper we focus on the psychometric testing element of the overall selection process. We wanted to test whether cultural factors may be driving poorer performance in the SJT, and whether having a first language other than English affects performance in the time-pressured WGCTAII assessment.

For peer review only - http://bmjopen.bmj.com/site/about/guidelines.xhtml
For peer review only

METHODS (461 words)

Data sources

A partially redacted dataset for those applying for training places in 2021[a] was extracted from the application system ("Oriel") by Health Education England East Midlands (HEEEM). This dataset covered applicant demography (sex, age group and ethnicity), professional background and progression through the selection process. For the first time in 2021, an optional enhanced set of questions were posed to candidates that sought to capture information about sociocultural background (Box 2). The data were passed to the research team, who then cleaned and coded the data for analysis.

Patient and public involvement

No patients involved.

[a] The year described relates to the August in which successful applicants would take up the role. The recruitment cycle begins the preceding November, such that the year being analysed includes those who began their application in November 2020 and potentially were selected to take up their post in August 2021.

For peer review only - http://bmjopen.bmj.com/site/about/guidelines.xhtml

Question	Response options	Source / basis of question
**What is the highest level of qualifications achieved by either of your parent(s) or guardian(s) by the time you were 18?**	▪ Degree level or Degree equivalent or above (for example first or higher degrees, postgraduate diplomas, NVQ/SVQ level 4 or 5, etc)<br>▪ Qualifications below degree level (for example an A-level, SCE Higher, GCSE, O-level, SCE Standard/Ordinary, NVQ/SVQ, BTEC, etc)<br>▪ No qualifications<br>▪ Do not know or cannot remember<br>▪ Prefer not to say<br>▪ Not applicable	The question is drawn from UK Government) guidance[23] on valid measures of socioeconomic status. More information can be found at: https://assets.publishing.service.gov.uk/government/uploads/system/uploads/attachment_data/file/713739/Annex_A-_Evaluation_of_measures_of_Socio-economic_background.pdf
**What is your main language?**	▪ English<br>▪ Welsh<br>▪ Arabic<br>▪ Bengali<br>▪ Chinese - Cantonese<br>▪ Chinese - Mandarin<br>▪ French<br>▪ German<br>▪ Gujurati<br>▪ Portuguese<br>▪ Punjabi<br>▪ Spanish<br>▪ Urdu<br>▪ Other: [Please complete]	The question is a repeat of the question asked in the UK (national) Census from 2011,[24] which drew on a broad base of stakeholder testing and validation. More information can be found at: https://www.ons.gov.uk/file?uri=/census/2011census/howourcensusworks/howweplannedthe2011census/questionnairedevelopment/finalisingthe2011questionnaire/final-recommended-questions-2011-language_tcm77-183991.pdf

Box 1. Enhanced data collection for Public Health Specialty Recruitment, 2021 recruitment round.

Professional background was assumed based on the eligibility criteria applied by each candidate: whether medical or BOTM. Additionally, we reviewed the degree awarding institution for all applicants who applied through the medical route to sort this group into UK trained medical doctors and those who had trained outside the UK (termed International Medical Graduates, IMGs). Every year, a small number of people with a primary medical qualification apply through the BOTM eligibility criteria. Applicants who hold a primary medical qualification but do not hold UK medical registration and a licence to practise may apply through the BOTM route. It is therefore possible that some doctors, including IMGs, applied through this route, but their identity was not captured from our process, resulting in them being labelled as BOTM.

Data processing

For peer review only - http://bmjopen.bmj.com/site/about/guidelines.xhtml

Data processing and analysis was undertaken in STATA SE 17.0 for Mac. The primary outcome for the analyses was ‘passing AC’ defined as having achieved a minimum threshold score in each of the three psychometric assessments. Sub-analyses were also undertaken (and are reported) for each psychometric test separately. Due to the comparatively small number of black and minority ethnic candidates, the ethnicity categories were aggregated upwards to create sufficiently large groups for analysis. Responses to the two new optional sociocultural questions (Box 1) were aggregated into: “Parental Degree” or “No parental degree”; and “English as main language” and “Not English as main language”.

Analytical approach

Descriptive analysis of passing the Assessment Centre and its constituent parts was undertaken with counts and percentages. Logistic regression was used for both univariable and multivariable analysis producing odds ratios (OR) and adjusted odds ratios (aOR) with 95% confidence intervals (CI), assuming two-tailed testing with an alpha of 0.05. Applicants for whom demographic data were missing are presented in descriptive analyses with count data but censored in regression analyses. Descriptive analyses where counts are <5 have been masked to reduce the risk of deductive disclosure and maintain privacy.

For peer review only

For peer review only - http://bmjopen.bmj.com/site/about/guidelines.xhtml

RESULTS (609 words)

There were 963 applicants for Public Health Specialty Recruitment in the 2021 round, of which 629 (65.3%) applicants passed eligibility checking and attended the Assessment Centre; the results presented are for these candidates only.

Of the candidates attending the Assessment Centre, approximately two-thirds were female (65.3%), just over half (57.7%) identified themselves as coming from white backgrounds (Table 1) and the median age was 32 years. In terms of professional background, 53.6% of AC attendees came from backgrounds other than medicine, while 11.6% and 34.8% were identified as coming from IMG and UK medical backgrounds respectively. The response rate to the enhanced question set (for those attending the Assessment Centre) was 96.0% in relation to parental qualification, but only 76.3% in relation to main language. Of those responding to these optional questions, 37.6% reported their parent or carer holding a degree-level qualification by the time they were 18 years old, and 93.5% reported their main language as being English.

Of those attending the Assessment Centre, 357 (56.8%) scored sufficiently well across the three assessments to 'pass' it.

For peer review only - http://bmjopen.bmj.com/site/about/guidelines.xhtml

Table 1. Descriptive analysis of demographic characteristics of candidates attending Assessment Centre 2021, with sub-analysis of Assessment Centre outcome (count and percentage).

	All candidates sitting AC n (%)	Candidates failing AC n (%)	Candidates passing AC n (%)
**Total [%]**	629	272 [43.2]	357 [56.8]
**Sex**			
- Male	199 (31.6)	89 (32.7)	110 (30.8)
- Female	411 (65.3)	177 (65.1)	234 (65.6)
- Other or not known	19 (3.0)	6 (2.2)	13 (3.6)
**Ethnic Group**			
- White British	304 (48.3)	98 (36.0)	206 (57.7)
- White Other	59 (9.4)	24 (8.8)	35 (9.8)
- Black	56 (8.9)	41 (15.1)	15 (4.2)
- Asian	114 (18.1)	65 (23.9)	49 (13.7)
- Mixed	32 (5.1)	12 (4.4)	20 (5.6)
- Chinese	11 (1.8)	7 (2.6)	4 (1.1)
- Other	15 (2.4)	8 (2.9)	7 (2.0)
- Not disclosed	38 (6.0)	17 (6.3)	21 (5.9)
**Age in years**			
- ≤29	209 (32.2)	61 (22.4)	148 (41.5)
- 30-34	181 (28.8)	77 (28.3)	104 (29.1)
- 35-39	96 (15.3)	53 (19.5)	43 (12.0)
- 40-44	70 (11.1)	42 (15.4)	28 (7.9)
- 45-49	33 (5.3)	21 (7.7)	12 (3.4)
- ≥50	9 (1.4)	5 (1.8)	4 (1.1)
- Not known	31 (4.9)	13 (4.8)	18 (5.0)
**Parent or carer highest qualification**			
- Degree	227 (36.1)	105 (38.6)	122 (34.2)
- No degree	377 (59.9)	159 (58.5)	218 (61.1)
- Not known	25 (4.0)	8 (2.9)	17 (4.8)
**Main language**			
- English	449 (71.4)	183 (67.3)	266 (74.5)
- Not English	31 (4.9)	18 (6.6)	13 (3.6)
- Not known	149 (23.7)	71 (26.1)	78 (21.9)
**Professional background**			
- BOTM	337 (53.6)	176 (64.7)	161 (45.1)
- IMG	73 (11.6)	57 (21.0)	16 (4.5)
- UK Medical	219 (34.8)	39 (14.3)	180 (50.4)

For peer review only

Breaking down the applicants by professional background (Table 2), female applicants outnumbered males across the groups but particularly so in the BOTM group (75.2% versus 25.0%). The median age was five years greater for BOTM candidates than among those from the UK medical graduate group. The BOTM group was more socioeconomically diverse than the two medical groupings with 41.6% (of responding applicants) reporting a parent or carer without a degree.

For peer review only - http://bmjopen.bmj.com/site/about/guidelines.xhtml

Table 2. Descriptive analysis of demographic characteristics of candidates by professional background in Assessment Centre 2021 (count and percentage).

	**All candidates from backgrounds other than medicine n (%)**	**All international medical graduate candidates n (%)**	**All UK medical graduate candidates n (%)**
**Total [%]**	337	73	219
**Sex**			
- Male	82 (24.8)	31 (43.1)	86 (41.3)
- Female	248 (75.2)	41 (56.9)	122 (58.7)
**Ethnic Group**			
- White British	191 (58.6)	<5	111 (55.0)
- White Other	30 (9.2)	13 (20.0)	16 (8.0)
- Black	24 (7.4)	23 (35.4)	9 (4.5)
- Asian	52 (16.0)	18 (27.7)	44 (22.0)
- Mixed	17 (5.2)	5 (7.7)	10 (5.0)
- Chinese	5 (1.5)	<5	5 (2.5)
- Other	7 (2.2)	<5	5 (2.5)
**Median age in years [interquartile range]**	34 [31-41]	31 [29-36]	29 [27-31]
**Parent or carer highest qualification**			
- Degree	192 (58.4)	50 (72.5)	135 (65.5)
- No degree	137 (41.6)	19 (27.5)	71 (34.5)
**Main language**			
- English	252 (95.8)	41 (71.9)	156 (97.5)
- Not English	11 (4.2)	16 (28.1)	<5

Logistic regression analyses (Table 3) suggested that that sex, age and main language were not statistically associated with passing the AC (termed hereafter 'progression'). However, there was statistically significant lower progression among applicants from black backgrounds (aOR 0.19, 95% CI 0.07 to 0.47) and Asian backgrounds (aOR 0.36, 95% CI 0.19-0.66). The association of lower progression probability with advancing age was not statistically significant after adjustment.

Professional background was also strongly associated with progression: compared to UK medical graduates, IMGs were more than 90% less likely to progress (aOR 0.07, 95% CI 0.03 to 0.17) and BOTM candidates approximately 80% less likely (aOR 0.21, 95% CI 0.11 to 0.39). Subgroup analysis of UK medical graduates only, where ethnicity was recorded (n = 200), showed a 14.2% percentage point gap between white British candidates (89.2% progressed) and those who were not white British (75.0%, p = 0.003).

For peer review only - http://bmjopen.bmj.com/site/about/guidelines.xhtml
For peer review only

Table 3. Univariable and multivariable logistic regression of demographic associations of candidates passing AC (odds ratios, adjusted odds ratios, 95% confidence intervals and p-values), with sub-group analysis of UK trained medical applicants (count, percentage and χ^2 p-value).

	**Odds ratio (95% CI)**	**p-value**	**Adjusted odds ratio* (95% CI)**	**p-value**
**Sex**				
- Male	Ref		Ref	
- Female	1.07 (0.76-1.50)	0.70	1.20 (0.73-1.97)	0.46
**Ethnic Group**				
- White British	Ref		Ref	
- White Other	0.69 (0.39 to 1.23)	0.21	1.10 (0.50 to 2.46)	0.89
- Black	0.17 (0.09 to 0.33)	<0.001	0.19 (0.07 to 0.47)	<0.001
- Asian	0.36 (0.23 to 0.56)	<0.001	0.36 (0.19 to 0.66)	<0.001
- Mixed	0.79 (0.37 to 1.69)	0.55	1.35 (0.49 to 3.69)	0.56
- Chinese	0.27 (0.08 to 0.95)	0.04	0.20 (0.03 to 1.58)	0.13
- Other	0.42 (0.15 to 1.18)	0.10	0.45 (0.11 to 1.80)	0.26
**Age in years****	0.92 (0.90 to 0.95)	<0.001	0.96 (0.93 to 1.00)	0.07
**Parent or carer highest qualification**				
- No degree	Ref		Ref	
- Degree	1.18 (0.85 to 1.64)	0.32	1.55 (0.99 to 2.43)	0.06
**Main language**				
- English	Ref		Ref	
- Not English	0.50 (0.34 to 1.04)	0.06	1.37 (0.52 to 3.67)	0.52
**Professional background**				
- BOTM	0.20 (0.13 to 0.30)	<0.001	0.21 (0.11 to 0.39)	<0.001
- IMG	0.06 (0.03 to 0.12)	<0.001	0.07 (0.03 to 0.17)	<0.001
- UK Medical	Ref		Ref	
Subgroup analysis of UK trained medical applicants only	*Passed AC* **n**	**(%)**	**χ^2 p-value**	
- White British (n = 111)	99	(89.2)	0.003	
- Ethnic minority (n = 89)	81	(75.0)		

* - adjustment includes sex, ethnic group, age, parent or carer degree status, first language and professional background.
** - age as a continuous variable in years.

Descriptive and inferential analysis of the RANRA, Watson-Glaser and SJT instruments separately demonstrated broadly similar results (Supplementary, Tables S1-3). The highest pass rate (79.3%) was achieved on the RANRA (numerical reasoning) assessment, followed by Watson-Glaser (74.1%) then SJT (73.9%).

Candidates from black backgrounds (black African, black Caribbean and black other) returned the lowest pass rates across all three instruments, followed by those from Asian backgrounds (Indian, Pakistani, Bangladeshi and other Asian). The performance gap by for those applicants from black backgrounds was

For peer review only - http://bmjopen.bmj.com/site/about/guidelines.xhtml
For peer review only

smallest on the SJT. The 'other' ethnic group also returned lower scores in univariable regression but after adjustment this was not the case. Younger age was associated with higher probability of progression – but the association was attenuated to a borderline finding following adjustment. UK medical graduates were significantly more likely to pass than the other two professional groups, with IMGs performing with the lowest scores overall. UK medical graduates showed the greatest performance gap with BOTM applicants in the RANRA (mean progression 95.4% versus 74.5%) and the SJT (mean progression 93.2% versus 67.4%).

Parental qualification was broadly not associated with performance (except weakly following adjustment in Watson-Glaser). Univariable regression suggested that main language other than English might be associated with lower scores across instruments; however, these associations were attenuated following adjustment in the multivariable regression.

For peer review only

For peer review only - http://bmjopen.bmj.com/site/about/guidelines.xhtml

DISCUSSION (1368 words)

Statement of principal findings

Coming from a black ethnic group, Asian ethnic group, or from a non-UK medical graduate background were all independently predictive of poorer performance across all three elements of the UK psychometric tests used in Public Health Specialty Recruitment in 2021. We detected no consistent pattern of lower-scoring performance associated with English as a second language, or from socioeconomic background (as measured by parental qualification). Increasing age may be associated with poorer performance, but some of the previously-noted performance gap appeared to be accounted for by confounding (possibly due to medical applicants, who perform better overall, tending to be younger). Yet even within the UK-trained medical cohort of applicants, there was differential attainment by ethnicity.

The hypothesis, outlined in our previous paper,[19] that cultural factors (including first language) might be causing poorer performance in the SJT the highly time-pressured Watson-Glaser were not supported by the analysis. We also hypothesised that the use of SJTs in other medical application processes might have conferred an advantage to UK medical graduates: yet the performance difference in the SJT was not substantially different to the gap observed in the RANRA. Instead, we observed consistent performance across all three instruments, with significantly lower performance by candidates from black and Asian backgrounds. Black candidates were 80% less likely than white British applicants to pass the Assessment Centre in 2021, and Asian candidates 60% less likely.

That these differences persisted even after adjustment for professional background and sub-categorisation of international medical graduates is deeply concerning. A further striking finding is the 90% differential attainment gap we observed between international medical graduates when compared against UK medical graduates. Once again, this difference persisted after multivariable adjustment.

Each of these three psychometric tests was developed and validated independently. Each test is designed to test a separate aspect of potential suitability for Public Health training. Yet similar patterns of differential attainment are observed across all three. Together, these findings add to the literature that suggests differential attainment is present across a wide range of psychometric tests.[25]

For peer review only - http://bmjopen.bmj.com/site/about/guidelines.xhtml
For peer review only

The earlier working hypothesis that age was likely confounded by professional background appears broadly justified although there remains some residual association after adjustment. The wider distribution of ages in the BOTM group may explain some of this difference. Although the two medical groups were socioeconomically more advantaged overall than the BOTM group, it was notable that the socioeconomic metric was not strongly associated with progression. However, the cohort entering the process appears more homogeneously drawn from higher socioeconomic groups than the UK population average. Accordingly, a type II error cannot be ruled out.

Strengths and weaknesses of the study

To our knowledge, this is the first study to systematically analyse differential attainment in a UK national selection process: while other analyses have made high-level comparisons, this study has examined in detail the intricacies of a single large-scale selection process and sought to anticipate confounding through the use of bespoke sociocultural questions, prospectively integrated into the process. Our findings have important implications for both Public Health as a specialty in the UK, and for the many other medical specialties that employ psychometric testing in their selection processes.

Among the study's weaknesses are the single-year nature of the dataset, and that ethnic groupings had to be aggregated to mitigate the analytical risk arising from small numbers. Accordingly, Type I and Type II errors cannot be ruled out. However, the consistency and strength of the findings around ethnicity and professional background suggest that a Type I error is highly unlikely in this context. The application of the sociocultural questions, while drawn from best available evidence on the measurement of socioeconomic status, may have been insufficient to detect differences, and the concept of socioeconomic status is complex. The response rate on parental qualification (at 96.0%) was very high; for the language question response rate was lower at 76.3%. The reason for the differential response rate on two sequential questions is unclear.

Strengths and weaknesses in other studies, discussing important differences in results

As already noted, in the context of the published literature, these differential attainment gaps are not as surprising as they might at first appear.[2] There is already extensive evidence of differential attainment relating to ethnicity across medical training in the UK.[26] Likewise, the broader evidence base on psychometric testing

For peer review only - http://bmjopen.bmj.com/site/about/guidelines.xhtml

shows relatively consistent findings that older and ethnic minority candidates generally perform less well on standardised testing.[25] While there remains some uncertainty over the precise drivers, possible explanations include specific barriers, such as differential access to networks, familiarity with psychometric testing,[27] test taker perception,[25] stereotype threat,[28] through to systemic disadvantage and structural racism occurring through the life-course.

Meaning of the study: possible explanations and implications for clinicians and policymakers

Due to Public Health recruitment drawing on a more diverse candidate pool than most other medical specialties (that require a primary medical qualification), comparing Public Health recruitment with other national programmes is difficult. Successful completion of medical school and foundation training is likely to create a more homogeneous and high-performing cohort of applicants. However, this study has confirmed that the observed differential attainment cannot be attributed to confounding alone: differential attainment was observed even within the UK medical graduate group. Instead, it appears that the system is unfairly discriminating against certain candidates.

While the status quo is unacceptable, we recognise the validity of the current recruitment process which has demonstrated that the candidates selected in the current process progress well in later Public Health training.[16] However there is some potential circularity to such a validation: that by selecting out people (through differential attainment) before training scheme entry, the trainees who do proceed are less subject to differential attainment later, leading to the appearance of a valid process. Differential attainment has been seen in postgraduate training in other specialties.[10] Any policy responses to this analysis need to preserve the strengths of the current system. Reducing a cohort of applicants from approximately 1000 to 100 needs a scalable, objective and practicable approach, and there is little doubt that the current system achieves some, if not most, of those objectives, at least in part.

In our preceding paper,[19] we outlined some of the potential responses to apparent differential attainment in the recruitment process more generally. In the context of these findings from the Assessment Centre stage specifically, the potential response options include a comprehensive review of the psychometric tests used to devise an assessment process, additional support for candidates from disadvantaged groups, or more affirmative action approaches that could see different thresholds set for different groups of candidates.

For peer review only - http://bmjopen.bmj.com/site/about/guidelines.xhtml

The implications of these findings reach well beyond Public Health. While most national recruitment processes draw only on medical graduates, the apparent effect of ethnicity among UK medical graduates, coupled with the apparent gap among international medical graduates, has important ramifications for all medical selection processes that are using psychometric tests. We propose that the assumption for selector teams must now be that there is differential attainment in all psychometric test instruments until proven otherwise. We encourage other specialties to explore the data on their own processes and to integrate prospective monitoring processes such as the sociocultural questions that we have employed in Public Health. Such efforts are imperative if we are to mitigate the risk of group-think perpetuating the selection of people who think like the 'establishment'.

Unanswered questions and future research

This study has identified differential attainment in the current national selection process for Public Health. And while it increasingly appears that differential attainment is a feature of psychometric testing in its present form, we are unable to clearly recommend mitigation that would be both simple and uncontentious. While there is a need for additional research, as a medical profession we must be careful to avoid additional analysis becoming a rationale for delaying our response.

Conclusions

All doctors and Public Health professionals need to have confidence in national systems of selection. We need a senior medical and public health workforce that better reflects the populations from which it is drawn and which they seek to serve. Public Health is a specialty that values diversity and promotes equity of opportunity. It is vital that we create a fairer future for doctors, health care and policy professionals.

For peer review only - http://bmjopen.bmj.com/site/about/guidelines.xhtml

Funding

Imperial College London is grateful for support from the NW London NIHR Applied Research Collaboration. The views expressed in this publication are those of the authors and not necessarily those of the NIHR or the Department of Health and Social Care.

Statement of involvement and engagement

Neither patients nor public were involved in the design, conduct, reporting or dissemination of this work.

Data sharing statement

The dataset was provided to the research team by Health Education England East Midlands for the analysis. Requests to access and analyse this dataset should be made to Health Education England East Midlands (https://www.eastmidlandsdeanery.nhs.uk/contact_us).

Competing interests

RJP is a Fellow of the Faculty of Public Health and former Technical Assessment Lead within the national Public Health Recruitment Executive Group hosted by Health Education England. FB is a Member of the Faculty of Public Health and a member of the Faculty's Special Interest Group in Equality and Diversity. FB is also a Specialty Registrar in Public Health. AM is a Fellow of the Faculty of Public Health. MR is a Fellow of the Faculty of Public Health and former Medical Advisor to NHS England on Workforce Race Equality.

Contributorship statement

RJP and FB conceived the study and undertook the analyses that are presented in this paper. RJP drafted the original version of this manuscript. FB, GS, AM and MR reviewed and revised the draft. MR is the guarantor of this paper.

Ethics approval statement

The analyses are evaluation and the data were taken from an administrative dataset. Permission was granted by the data owner to use these data for the explicit analytical purposes of this paper, but these analyses and approach were not anticipated at the time that the data were collected.

For peer review only

For peer review only - http://bmjopen.bmj.com/site/about/guidelines.xhtml

References

1. Nagpaul, C. Racism is wrecking the lives of doctors, affecting patient care, and jeopardising services. *BMJ* **377**, o1515 (2022).
2. Woolf, K. Differential attainment in medical education and training. *BMJ* **368**, (2020).
3. Patterson, F., Tiffin, P. A., Lopes, S. & Zibarras, L. Unpacking the dark variance of differential attainment on examinations in overseas graduates. *Med Educ* **52**, 736–746 (2018).
4. Tiffin, P. A. & Paton, L. W. Differential attainment in the MRCPsych according to ethnicity and place of qualification between 2013 and 2018: a UK cohort study. *Postgrad Med J* **97**, 764–776 (2021).
5. Kar, P. Partha Kar: Racism in the medical workforce—five initial steps. *BMJ* **377**, o1280 (2022).
6. Gravlee, C. C. How race becomes biology: embodiment of social inequality. *Am J Phys Anthropol* **139**, 47–57 (2009).
7. General Medical Council. What is differential attainment? https://www.gmc-uk.org/education/standards-guidance-and-curricula/projects/differential-attainment/what-is-differential-attainment (2022).
8. McManus, I. C., Dewberry, C., Nicholson, S. & Dowell, J. S. The UKCAT-12 study: Educational attainment, aptitude test performance, demographic and socio-economic contextual factors as predictors of first year outcome in a cross-sectional collaborative study of 12 UK medical schools. *BMC Med* **11**, (2013).
9. Hope, D. *et al.* Measuring differential attainment: a longitudinal analysis of assessment results for 1512 medical students at four Scottish medical schools. *BMJ Open* **11**, (2021).
10. Shah, R. & Ahluwalia, S. The challenges of understanding differential attainment in postgraduate medical education. *British Journal of General Practice* **69**, 426–427 (2019).
11. Royal College of Physicians, L. *2017 survey of medical certificate of completion of training (CCT) holders' career progression*. https://www.rcplondon.ac.uk/projects/outputs/2017-survey-medical-certificate-completion-training-cct-holders-career-progression (2018).
12. Powell, R. A. *et al.* Tackling racism in UK health research. *BMJ* **376**, (2022).
13. Consul, N., Strax, R., DeBenedectis, C. M. & Kagetsu, N. J. Mitigating Unconscious Bias in Recruitment and Hiring. *J Am Coll Radiol* **18**, 769–773 (2021).
14. Manchester tackles failure rate of Asian students. *BMJ* **310**, 209 (1995).

For peer review only - http://bmjopen.bmj.com/site/about/guidelines.xhtml

15. Koczwara, A. *et al.* Evaluating cognitive ability, knowledge tests and situational judgement tests for postgraduate selection. *Med Educ* **46**, 399–408 (2012).

16. Pashayan, N. *et al.* Evaluation of recruitment and selection for specialty training in public health: interim results of a prospective cohort study to measure the predictive validity of the selection process. *J Public Health (Bangkok)* **38**, e194–e200 (2016).

17. Work Psychology Group. *Multi-Specialty Recruitment Assessment (MSRA) - Test Blueprint & Information*. https://heeoe.hee.nhs.uk/sites/default/files/msra_test_blueprint_information_nov_2019.pdf (2019).

18. Tekkis, N. P. *et al.* The impact of the COVID-19 pandemic on UK medical education. A nationwide student survey. *Med Teach* (2021) doi:10.1080/0142159X.2021.1962835/SUPPL_FILE/IMTE_A_1962835_SM5137.DOCX.

19. Bury, F., Rao, M. & Pinder, R. Differential attainment in Public Health specialty training recruitment in the United Kingdom: an observational analysis of applicants from 2018 to 2020. *J Public Health (Bangkok)* **Submitted**, (2022).

20. Rust, J. *Advanced Numerical Reasoning Appraisal TM (ANRA) Manual*. https://docplayer.net/44427444-Rust-advanced-numerical-reasoning-appraisal.html (2006).

21. Watson, G. & Glaser, E. M. *Watson-Glaser TM II Critical Thinking Appraisal Technical Manual and User's Guide*. https://www.yumpu.com/en/document/read/41941997/watson-glaser-user-guide-and-technical-manual-talentlens (2010).

22. Iacobucci, G. Specialty training: ethnic minority doctors' reduced chance of being appointed is "unacceptable". *BMJ* **368**, m479 (2020).

23. *Annex A - Evaluation of measures of socio-economic background*. (2018).

24. Census 2011. *Final recommended questions for the 2011 Census in England and Wales, Language*. (2009).

25. Hough, L. M., Oswald, F. L. & Ployhart, R. E. Determinants, Detection and Amelioration of Adverse Impact in Personnel Selection Procedures: Issues, Evidence and Lessons Learned. *International Journal of Selection and Assessment* **9**, 152–194 (2001).

26. Linton, S. Taking the difference out of attainment. *BMJ* **368**, (2020).

For peer review only - http://bmjopen.bmj.com/site/about/guidelines.xhtml

27. Hinton, D. P. Uncovering the root causes of ethnic differences in ability testing: Differential test functioning, test familiarity and trait optimism as explanations of ethnic group differences. (Aston University, 2014).

28. Steele, C. M. A threat in the air. How stereotypes shape intellectual identity and performance. *Am Psychol* **52**, 613–629 (1997).

For peer review only

For peer review only - http://bmjopen.bmj.com/site/about/guidelines.xhtml

Differential attainment in specialty training recruitment in the United Kingdom: an observational analysis of the impact of psychometric testing assessment in Public Health postgraduate selection.

Submission for BMJ OPEN

STRICTLY CONFIDENTIAL

For peer review only

Authors

Richard J Pinder[1] **Corresponding author; richard.pinder@imperial.ac.uk**

ORCID: 0000-0002-7010-6009;

Fran Bury[1] ORCID: 0000-0002-6305-1994

Ganesh Sathyamoorthy[1,2, 3]

Azeem Majeed[1,3] ORCID: 0000-0002-2357-9858

Mala Rao[1,2,3] ORCID: 0000-0001-5504-6303

Affiliations

1. Department of Primary Care and Public Health, School of Public Health, Imperial College London
2. Ethnicity and Health Unit, Department of Primary Care and Public Health, Imperial College London
3. NIHR Applied Research Collaboration Northwest London

Key words

Public health, recruitment, medical training, ethnicity, differential attainment

SUPPLEMENTARY MATERIALS

For peer review only - http://bmjopen.bmj.com/site/about/guidelines.xhtml

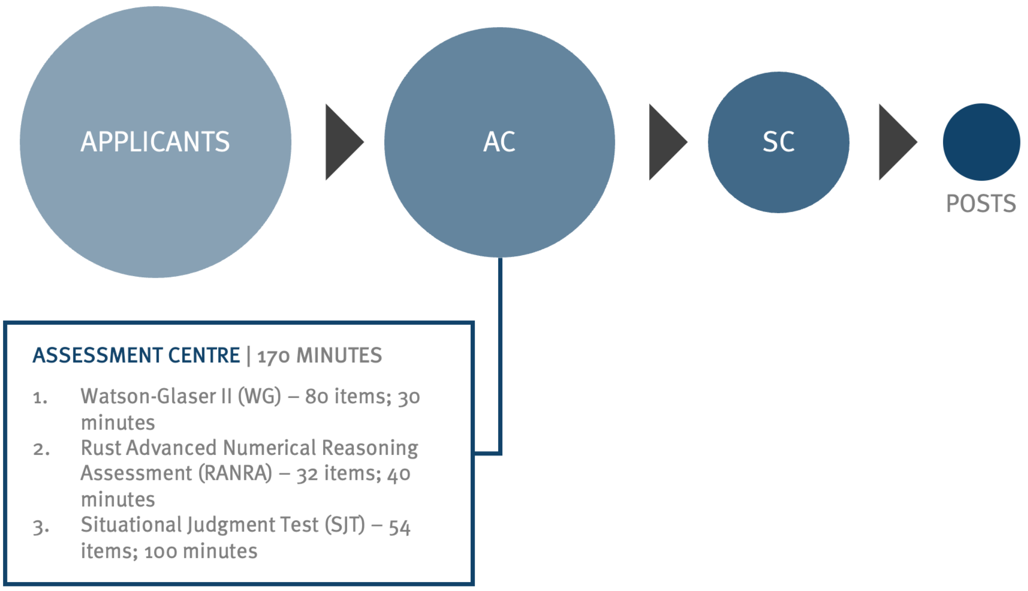

Supplementary Figure 1. Recruitment cycle diagram for Public Health specialty training, 2009-2020

For peer review only

For peer review only - http://bmjopen.bmj.com/site/about/guidelines.xhtml

Supplementary Table 1. Univariable and multivariable logistic regression of demographic associations of candidates passing RANRA (count, percentage, odds ratios, adjusted odds ratios, 95% confidence intervals and p-values).

	Passed RANRA (% of those sitting)	Odds ratio (95% CI)	p-value	Adjusted odds ratio* (95% CI)	p-value
**Overall**	499 (79.3)				
**Sex**					
- Male	157 (78.9)	Ref		Ref	
- Female	324 (78.8)	1.00 (0.66 to 1.51)	0.99	1.06 (0.59 to 1.90)	0.85
**Ethnic Group**					
- White British	270 (88.8)	Ref		Ref	
- White Other	47 (79.7)	0.49 (0.24 to 1.02)	0.06	0.90 (0.33 to 2.48)	0.85
- Black	31 (55.4)	0.16 (0.08 to 0.30)	<0.001	0.19 (0.08 to 0.44)	<0.001
- Asian	79 (69.3)	0.28 (0.17 to 0.49)	<0.001	0.35 (0.16 to 0.71)	<0.001
- Mixed	25 (78.1)	0.45 (0.18 to 1.11)	0.09	0.63 (0.20 to 1.99)	0.43
- Chinese	8 (72.7)	0.34 (0.08 to 1.33)	0.12	0.55 (0.05 to 6.21)	0.63
- Other	10 (66.7)	0.25 (0.08 to 0.78)	0.02	0.31 (0.07 to 1.36)	0.12
**Age in years****	Range: 24 to 55	0.92 (0.89 to 0.95)	<0.001	0.95 (0.91 to 0.99)	0.02
**Parent or carer highest qualification**					
- No degree	180 (79.3)	Ref		Ref	
- Degree	300 (79.6)	1.02 (0.68 to 1.53)	0.93	1.16 (0.67 to 1.77)	0.60
**Main language**					
- English	363 (80.9)	Ref		Ref	
- Not English	19 (61.3)	0.38 (0.18 to 0.80)	0.01	0.66 (0.25 to 1.77)	0.41
**Professional background**					
- BOTM	251 (74.5)	0.14 (0.07 to 0.28)	<0.001	0.18 (0.07 to 0.47)	<0.001
- IMG	39 (53.4)	0.05 (0.03 to 0.12)	<0.001	0.10 (0.04 to 0.32)	<0.001
- UK Medical	209 (95.4)	Ref		Ref	

* - adjustment includes sex, ethnic group, age, parent or carer degree status, main language and professional background.
** - age as a continuous variable in years.

For peer review only - http://bmjopen.bmj.com/site/about/guidelines.xhtml

Supplementary Table 2. Univariable and multivariable logistic regression of demographic associations of candidates passing Watson-Glaser (count, percentage, odds ratios, adjusted odds ratios, 95% confidence intervals and p-values).

	Passed WG (% of those sitting)	Odds ratio (95% CI)	p-value	Adjusted odds ratio* (95% CI)	p-value
**Overall**	466 (74.1)				
**Sex**					
- Male	140 (70.4)	Ref			
- Female	310 (75.4)	1.29 (0.89-1.89)	0.18	1.25 (0.73-2.17)	0.41
**Ethnic Group**					
- White British	255 (83.9)	Ref		Ref	
- White Other	47 (79.7)	0.75 (0.37-1.52)	0.43	1.22 (0.45-3.30)	0.70
- Black	26 (46.4)	0.17 (0.91-0.31)	<0.001	0.20 (0.09-0.46)	<0.001
- Asian	72 (63.2)	0.33 (0.20-0.54)	<0.001	0.30 (0.16-0.58)	<0.001
- Mixed	26 (81.3)	0.83 (0.33-2.13)	0.70	1.24 (0.36-4.30)	0.73
- Chinese	6 (54.6)	0.23 (0.68-0.79)	0.02	0.22 (0.03-1.74)	0.15
- Other	8 (53.3)	0.22 (0.08-0.63)	0.01	0.25 (0.06-1.00)	0.05
**Age in years****	Range: 24-54	0.94 (0.92-0.97)	<0.001	0.95 (0.92-1.00)	0.03
**Parent or carer highest qualification**					
- No degree	159 (70.0)	Ref		Ref	
- Degree	287 (76.1)	1.36 (0.94-1.97)	0.10	1.78 (1.07-2.98)	0.03
**Main language**					
- English	347 (77.3)	Ref		Ref	
- Not English	19 (61.3)	0.47 (0.22-0.99)	0.05	0.84 (0.31-2.23)	0.72
**Professional background**					
- BOTM	243 (72.1)	0.35 (0.22-0.56)	<0.001	0.39 (0.19-0.80)	0.01
- IMG	30 (41.1)	0.09 (0.05-0.17)	<0.001	0.13 (0.05-0.33)	<0.001
- UK Medical	193 (88.1)	Ref		Ref	

* - adjustment includes sex, ethnic group, age, parent or carer degree status, main language and professional background.
** - age as a continuous variable in years.

For peer review only

For peer review only - http://bmjopen.bmj.com/site/about/guidelines.xhtml

Supplementary Table 3. Univariable and multivariable logistic regression of demographic associations of candidates passing Situational Judgment Test (count, percentage, odds ratios, adjusted odds ratios, 95% confidence intervals and p-values).

	Passed SJT (% of those sitting)	Odds ratio (95% CI)	p-value	Adjusted odds ratio* (95% CI)	p-value
**Overall**	465 (73.9)				
**Sex**					
- Male	143 (71.9)	Ref		Ref	
- Female	307 (74.7)	1.16 (0.79-1.69)	0.46	1.59 (0.94-2.69)	0.09
**Ethnic Group**					
- White British	251 (82.6)	Ref		Ref	
- White Other	44 (74.6)	0.62 (0.32-1.19)	0.15	1.25 (0.49-3.21)	0.64
- Black	30 (53.6)	0.24 (0.13-0.45)	<0.001	0.39 (0.17-0.88)	0.02
- Asian	73 (64.0)	0.38 (0.23-0.61)	<0.001	0.38 (0.20-0.74)	0.01
- Mixed	22 (68.8)	0.46 (0.21-1.04)	0.06	0.74 (0.26-2.11)	0.57
- Chinese	7 (63.6)	0.37 (0.10-1.31)	0.12	0.26 (0.03-2.24)	0.22
- Other	9 (60.0)	0.32 (0.11-0.93)	0.04	0.30 (0.07-1.22)	0.09
**Age in years****	Range: 24-50	0.93 (0.90-0.95)		0.97 (0.93-1.01)	0.11
**Parent or carer highest qualification**					
- No degree	164 (72.3)	Ref		Ref	
- Degree	280 (74.3)	1.11 (0.77-1.61)	0.59	1.36 (0.83-2.23)	0.22
**First language**					
- English	339 (75.5)	Ref		Ref	
- Other	17 (54.8)	0.39 (0.19-0.83)	0.01	1.02 (0.68-2.71)	0.97
**Professional background**					
- BOTM	227 (67.4)	0.15 (0.09-0.27)	<0.001	0.16 (0.07-0.35)	<0.001
- IMG	34 (46.6)	0.06 (0.03-0.13)	<0.001	0.07 (0.03-0.19)	<0.001
- UK Medical	204 (93.2)	Ref		Ref	

* - adjustment includes sex, ethnic group, age, parent or carer degree status, main language and professional background.
** - age as a continuous variable in years.

For peer review only

STROBE Statement—checklist of items that should be included in reports of observational studies

	Item No	Recommendation	Page No
**Title and abstract**	1	(*a*) Indicate the study's design with a commonly used term in the title or the abstract	Yes, Page 1. "…an observational analysis…"
		(*b*) Provide in the abstract an informative and balanced summary of what was done and what was found	Yes, Page 2.
**Introduction**			
Background/rationale	2	Explain the scientific background and rationale for the investigation being reported	Yes, Page 4-5.
Objectives	3	State specific objectives, including any prespecified hypotheses	Yes, final two sentences; Page 5.
**Methods**			
Study design	4	Present key elements of study design early in the paper	Yes, paragraph 2, Page 8
Setting	5	Describe the setting, locations, and relevant dates, including periods of recruitment, exposure, follow-up, and data collection	Yes, Page 6 (including footnote [a]).
Participants	6	(*a*) *Cohort study*—Give the eligibility criteria, and the sources and methods of selection of participants. Describe methods of follow-up<br>*Case-control study*—Give the eligibility criteria, and the sources and methods of case ascertainment and control selection. Give the rationale for the choice of cases and controls<br>*Cross-sectional study*—Give the eligibility criteria, and the sources and methods of selection of participants	Observational study, technically prospective cohort by design. This criterion is not immediately relevant to this evaluation-driven approach. However eligibility criteria, sources and follow-up are described in the methods section.
		(*b*) *Cohort study*—For matched studies, give matching criteria and number of exposed and unexposed<br>*Case-control study*—For matched studies, give matching criteria and the number of controls per case	N/A
Variables	7	Clearly define all outcomes, exposures, predictors, potential confounders, and effect modifiers. Give diagnostic criteria, if applicable	Yes, Box 1 page 7. All elements described pages 7-8.
Data sources/ measurement	8*	For each variable of interest, give sources of data and details of methods of assessment (measurement). Describe	Yes. First paragraph, Page 6/

For peer review only - http://bmjopen.bmj.com/site/about/guidelines.xhtml

For peer review only

		comparability of assessment methods if there is more than one group	
Bias	9	Describe any efforts to address potential sources of bias	Yes. Regression approach outlined on page 8.
Study size	10	Explain how the study size was arrived at	Yes. First paragraph, page 9.
Quantitative variables	11	Explain how quantitative variables were handled in the analyses. If applicable, describe which groupings were chosen and why	Yes. Table 1, page 10.
Statistical methods	12	(*a*) Describe all statistical methods, including those used to control for confounding	Yes. Page 8.
		(*b*) Describe any methods used to examine subgroups and interactions	Yes. Table 2 legend explains the breakdown by professional grouping. Supplementary tables provide more granular analysis by psychometric instrument.
		(*c*) Explain how missing data were addressed	Yes. Page 8, paragraph 2.
		(*d*) *Cohort study*—If applicable, explain how loss to follow-up was addressed<br>*Case-control study*—If applicable, explain how matching of cases and controls was addressed<br>*Cross-sectional study*—If applicable, describe analytical methods taking account of sampling strategy	There was no loss to follow up as this was an observational analysis /evaluation with a complete data set.
		(*e*) Describe any sensitivity analyses	Provided through supplementary tables S1-S3.

Continued on next page

For peer review only

**Results**			
Participants	13*	(a) Report numbers of individuals at each stage of study—eg numbers potentially eligible, examined for eligibility, confirmed eligible, included in the study, completing follow-up, and analysed	Yes. Paragraph 1, Page 9.
		(b) Give reasons for non-participation at each stage	Yes. Not applicable as selection process and this is an analysis of a single stage.
		(c) Consider use of a flow diagram	This was not felt necessary, but can be added at the request of the editor.
Descriptive data	14*	(a) Give characteristics of study participants (eg demographic, clinical, social) and information on exposures and potential confounders	Yes. Table 1 (Page 10) provides breakdown, and Table 2 provides additional data for confounding by professional background / route of application.
		(b) Indicate number of participants with missing data for each variable of interest	Yes; "Not known" is provided as a line for each variable in Table 1.
		(c) *Cohort study*—Summarise follow-up time (eg, average and total amount)	N/A
Outcome data	15*	*Cohort study*—Report numbers of outcome events or summary measures over time	Provided in final column of Table 1.
		Case-control study—Report numbers in each exposure category, or summary measures of exposure	N/A
		Cross-sectional study—Report numbers of outcome events or summary measures	N/A
Main results	16	(*a*) Give unadjusted estimates and, if applicable, confounder-adjusted estimates and their precision (eg, 95% confidence interval). Make clear which confounders were adjusted for and why they were included	Yes. Table 3 – point estimate odds ratio with 95% confidence intervals – for both univariable and multivariable analyses.
		(*b*) Report category boundaries when continuous variables were categorized	N/A
		(*c*) If relevant, consider translating estimates of relative risk into absolute risk for a meaningful time period	N/A
Other analyses	17	Report other analyses done—eg analyses of subgroups and interactions, and sensitivity analyses	Yes. Supplementary tables S1-S3 provide more granular analysis.
**Discussion**			
Key results	18	Summarise key results with reference to study objectives	Yes – Paragraph 1, page 14.
Limitations	19	Discuss limitations of the study, taking into account sources of potential bias or imprecision. Discuss both direction and magnitude of any potential bias	Yes, Paragraphs 2 and 3, page 15.
Interpretation	20	Give a cautious overall interpretation of results considering objectives, limitations, multiplicity of analyses, results from similar studies, and other relevant evidence	Yes, throughout Discussion.

For peer review only - http://bmjopen.bmj.com/site/about/guidelines.xhtml

For peer review only

Generalisability	21	Discuss the generalisability (external validity) of the study results	Yes. Paragraph 1, page 17.
**Other information**			
Funding	22	Give the source of funding and the role of the funders for the present study and, if applicable, for the original study on which the present article is based	Yes – in acknowledgements, page 18.

*Give information separately for cases and controls in case-control studies and, if applicable, for exposed and unexposed groups in cohort and cross-sectional studies.

Note: An Explanation and Elaboration article discusses each checklist item and gives methodological background and published examples of transparent reporting. The STROBE checklist is best used in conjunction with this article (freely available on the Web sites of PLoS Medicine at http://www.plosmedicine.org/, Annals of Internal Medicine at http://www.annals.org/, and Epidemiology at http://www.epidem.com/). Information on the STROBE Initiative is available at www.strobe-statement.org.

For peer review only

For peer review only - http://bmjopen.bmj.com/site/about/guidelines.xhtml

BMJ Open

Differential attainment in specialty training recruitment in the United Kingdom: an observational analysis of the impact of psychometric testing assessment in Public Health postgraduate selection.

Journal:	*BMJ Open*
Manuscript ID	bmjopen-2022-069738.R1
Article Type:	Original research
Date Submitted by the Author:	07-Feb-2023
Complete List of Authors:	Pinder, Richard; Imperial College London School of Public Health, Department of Primary Care and Public Health<br>Bury, Fran; Imperial College London<br>Sathyamoorthy, Ganesh; Imperial College London, NIHR Northwest London Applied Research Collaboration<br>Majeed, Azeem; Imperial College London, Primary Care<br>Rao, Mala; Imperial College London, Department of Public Health
**Primary Subject Heading**:	Public health
Secondary Subject Heading:	Medical education and training
Keywords:	Human resource management < HEALTH SERVICES ADMINISTRATION & MANAGEMENT, PUBLIC HEALTH, Health policy < HEALTH SERVICES ADMINISTRATION & MANAGEMENT

SCHOLARONE™
Manuscripts

I, the Submitting Author has the right to grant and does grant on behalf of all authors of the Work (as defined in the below author licence), an exclusive licence and/or a non-exclusive licence for contributions from authors who are: i) UK Crown employees; ii) where BMJ has agreed a CC-BY licence shall apply, and/or iii) in accordance with the terms applicable for US Federal Government officers or employees acting as part of their official duties; on a worldwide, perpetual, irrevocable, royalty-free basis to BMJ Publishing Group Ltd ("BMJ") its licensees and where the relevant Journal is co-owned by BMJ to the co-owners of the Journal, to publish the Work in this journal and any other BMJ products and to exploit all rights, as set out in our licence.

The Submitting Author accepts and understands that any supply made under these terms is made by BMJ to the Submitting Author unless you are acting as an employee on behalf of your employer or a postgraduate student of an affiliated institution which is paying any applicable article publishing charge ("APC") for Open Access articles. Where the Submitting Author wishes to make the Work available on an Open Access basis (and intends to pay the relevant APC), the terms of reuse of such Open Access shall be governed by a Creative Commons licence – details of these licences and which Creative Commons licence will apply to this Work are set out in our licence referred to above.

Other than as permitted in any relevant BMJ Author's Self Archiving Policies, I confirm this Work has not been accepted for publication elsewhere, is not being considered for publication elsewhere and does not duplicate material already published. I confirm all authors consent to publication of this Work and authorise the granting of this licence.

For peer review only

Differential attainment in specialty training recruitment in the United Kingdom: an observational analysis of the impact of psychometric testing assessment in Public Health postgraduate selection.

Submission for BMJ OPEN

Revision 1

STRICTLY CONFIDENTIAL

Authors

Richard J Pinder[1] **Corresponding author; richard.pinder@imperial.ac.uk**

ORCID: 0000-0002-7010-6009;

Fran Bury[1] ORCID: 0000-0002-6305-1994

Ganesh Sathyamoorthy[1,2, 3]

Azeem Majeed[1,3] ORCID: 0000-0002-2357-9858

Mala Rao[1,2,3] ORCID: 0000-0001-5504-6303

Affiliations

1. Department of Primary Care and Public Health, School of Public Health, Imperial College London
2. Ethnicity and Health Unit, Department of Primary Care and Public Health, Imperial College London
3. NIHR Applied Research Collaboration Northwest London

Key words

Public health, recruitment, medical training, ethnicity, differential attainment

3580 words excluding abstract, 1 box and 3 tables.
Supplementary data: 1 figure and 3 tables.

For peer review only - http://bmjopen.bmj.com/site/about/guidelines.xhtml

ABSTRACT (280 words)

Objectives

To determine how current psychometric testing approaches used in selection to postgraduate training in UK Public Health is associated with socioeconomic and sociocultural background of applicants (including ethnicity).

Design

Observational study using contemporaneous data collected during recruitment and psychometric test scores.

Setting

Assessment Centre of UK national Public Health recruitment for postgraduate Public Health training. The Assessment Centre element of selection comprises three psychometric assessments: Rust Advanced Numerical Reasoning (RANRA), Watson Glaser Critical Thinking Assessment II (WGCTAII) and Public Health situational judgment test (SJT).

Participants

629 applicants completed the Assessment Centre in 2021. 219 (34.8%) were UK medical graduates, 73 (11.46%) were international medical graduates, and 337 (53.6%) were from backgrounds other than medicine.

Main outcome measure

Multivariable adjusted progression statistics in the form of adjusted odds ratio (aOR), accounting for age, sex, ethnicity, professional background and surrogate measures of familial socioeconomic and sociocultural status.

Results

357 (56.8%) candidates passed all three psychometric tests . Candidate characteristics negatively associated with progression were black ethnicity (aOR 0.19, 0.08 to 0.44), Asian ethnicity (aOR 0.35, 0.16-0.71) and coming from a non-UK medical graduate background (aOR 0.05, 0.03 to 0.12); similar differential attainment was observed in each of the psychometric tests. Even within the UK-trained medical cohort, candidates from white British backgrounds were more likely to progress than those from ethnic minorities (89.2% versus 75.0%, p=0.003).

For peer review only

For peer review only - http://bmjopen.bmj.com/site/about/guidelines.xhtml

Conclusion

Although perceived to mitigate the risks of conscious and unconscious bias in selection to medical postgraduate training, these psychometric tests demonstrate unexplained variation that suggests differential attainment. Other specialities should enhance their data collection to evaluate the impact of differential attainment on current selection processes and take forward opportunities to mitigate differential attainment where possible.

STRENGTHS AND LIMITATIONS OF THIS STUDY

- This study analyses a complete annual cohort of applicants for UK Public Health specialty training in 2021 to identify the extent and impact of differential attainment arising in the psychometric testing component of selection.
- The methodology employs an enhanced and prospectively-collected set of questions that explore sociocultural background of applicants, as well as categorising UK medical graduates, international medical graduates and candidates from backgrounds other than medicine.
- Socioeconomic disadvantage and intersectionality are inherently complex and difficult to quantify; we cannot exclude the possibility of other potential confounders.

For peer review only

For peer review only - http://bmjopen.bmj.com/site/about/guidelines.xhtml

INTRODUCTION (796 words)

Over recent years, awareness of the challenges facing doctors and other healthcare workers from ethnic minority backgrounds has increased.[1] The inequity of opportunity and outcome is driven by myriad factors, including: barriers to professional progression;[2–4] more active forms of racism from patients, colleagues and institutions;[5] and the interaction with other social categorisations (so-called intersectionality).[6] Differential attainment describes a gap in attainment levels between different demographic groups, including by protected characteristics.[7] Differential attainment has been observed in medical school selection,[8,9] during undergraduate medical training,[10] in postgraduate training[11] in senior appointments,[12] and in the field of medical research.[13]

Like other employment sectors, the medical profession internationally has tried to mitigate unconscious bias arising during recruitment.[14] As part of the historical shift from resource-intensive interviews where unconscious (and potentially even conscious) bias has been suspected,[15] psychometric testing has been commonly perceived as more objective.[2] And while validation of psychometric testing in the context of United Kingdom (UK) General Practice recruitment[16] and Public Health recruitment[17] has been published, conceptual validity has been largely predicated on subsequent professional progression as opposed to investigating potential differential attainment.

The use and weighting accorded to psychometric tests for postgraduate medical recruitment the UK varies. The Multi-Specialty Recruitment Assessment[18] (MSRA) is a multi-instrument assessment of procedural and clinical knowledge[19] that began in general practice and which has also been used in ophthalmology and obstetrics and gynaecology for several years.[20] In 2022, around 10 UK specialties used MSRA as a means of shortlisting candidates.

Public Health is an unusual specialty that recruits both medical and non-medical candidates. The latter are referred to as candidates from *backgrounds other than medicine* (BOTM). The current three-stage design of postgraduate specialty recruitment for Public Health (Supplementary Figure 1) comprises eligibility checking, an assessment centre (AC) with three psychometric instruments, and a selection centre (SC); this process reduces approximately 1000 applicants to 650 applicants at AC and 216 applicants at SC.[17,21] This selection process preceded the introduction of the MSRA.

For peer review only - http://bmjopen.bmj.com/site/about/guidelines.xhtml

Due to the diversity of Public Health's applicant pool, MSRA (which assesses elements of clinical decision making) would not be a fair assessment tool. Instead, since 2009 Public Health has used two generic psychometric instruments that are used internationally in recruiting to corporate roles: Rust Advanced Numerical Reasoning Assessment[22] (RANRA) and Watson Glaser Critical Thinking Assessment II[23] (WGCTAII). A third bespoke situational judgment test (SJT) instrument is designed and implemented by Work Psychology Group with input from Public Health subject matter experts and was added to the process in 2011. The SJT provides a hypothetical scenario for a Public Health registrar to appraise and then rank in order of suitability their response from a list of stated actions. The three instruments are undertaken as a 170-minute computer-based test. Much-like MSRA, the Public Health AC is operated in multiple locations by the commercial testing company PearsonVue.

In 2020, the BMJ published a news story suggesting that for every specialty included, white candidates were more likely to be deemed appointable for specialty training than ethnic minority candidates.[24] Among the specialties considered, Public Health had the greatest differential between white and ethnic minority candidates in the proportion deemed appointable. Prompted by the article, this research was independently commissioned by the UK Public Health Recruitment Executive Group (REG, a committee of Health Education England) to determine whether some groups of candidates for Public Health specialty training were less likely to be successful in their applications than others, and to determine at which stage(s) of the process this occurs. The analysis, drafting and decision to submit this paper was undertaken exclusively by the authors. This study forms the second and final paper of the commissioned work.

Our preceding paper[21] analysed 2252 applicants over the three years 2018-20 inclusive of the three-stage process. Contrary to initial beliefs that the SC interviews might be the source of the differential attainment, the greatest differential attainment was associated with the psychometric stage of assessment.[21] Applicants from black backgrounds and Asian backgrounds, as well as those from BOTM were between 70% and 90% less likely to progress from the psychometric testing stage than white British candidates and medical candidates respectively. As a result of these observations, we considered residual confounding as a possible explanation, recognising our inability to disaggregate international medical graduates (who are less likely to be white British) as well as to detect other socioeconomic differences. Also of interest was whether the SJT

For peer review only - http://bmjopen.bmj.com/site/about/guidelines.xhtml

specifically might be contributing to differential attainment by assuming cultural understanding of the British workplace: for example in assessing etiquette around punctuality and other culturally nuanced professional behaviours. Finally, we sought to assess whether having a first language other than English affects performance in the time-pressured WGCTAII assessment.

METHODS (476 words)

Data sources

A partially redacted dataset (to comply with the principle of data minimisation[25]) for those applying for training places in 2021[a] was extracted from the application system ("Oriel") by Health Education England East Midlands (HEEEM). This dataset covered applicant demography (sex, age group and ethnicity), professional background and progression through the selection process. For the first time in 2021, an optional enhanced set of questions were posed to candidates that sought to capture information about sociocultural background (Box 1). The data were passed to the research team, who then cleaned and coded the data for analysis.

Patient and public involvement

No patients involved.

[a] The year described relates to the August in which successful applicants would take up the role. The recruitment cycle begins the preceding November, such that the year being analysed includes those who began their application in November 2020 and potentially were selected to take up their post in August 2021.

For peer review only - http://bmjopen.bmj.com/site/about/guidelines.xhtml

Question	Response options	Source / basis of question
**What is the highest level of qualifications achieved by either of your parent(s) or guardian(s) by the time you were 18?**	▪ Degree level or Degree equivalent or above (for example first or higher degrees, postgraduate diplomas, NVQ/SVQ level 4 or 5, etc)<br>▪ Qualifications below degree level (for example an A-level, SCE Higher, GCSE, O-level, SCE Standard/Ordinary, NVQ/SVQ, BTEC, etc)<br>▪ No qualifications<br>▪ Do not know or cannot remember<br>▪ Prefer not to say<br>▪ Not applicable	The question is drawn from UK Government) guidance[26] on valid measures of socioeconomic status. More information can be found at: https://assets.publishing.service.gov.uk/government/uploads/system/uploads/attachment_data/file/713739/Annex_A-_Evaluation_of_measures_of_Socio-economic_background.pdf
**What is your main language?**	▪ English<br>▪ Welsh<br>▪ Arabic<br>▪ Bengali<br>▪ Chinese - Cantonese<br>▪ Chinese - Mandarin<br>▪ French<br>▪ German<br>▪ Gujurati<br>▪ Portuguese<br>▪ Punjabi<br>▪ Spanish<br>▪ Urdu<br>▪ Other: [Please complete]	The question is a repeat of the question asked in the UK (national) Census from 2011,[27] which drew on a broad base of stakeholder testing and validation. More information can be found at: https://www.ons.gov.uk/file?uri=/census/2011census/howourcensusworks/howweplannedthe2011census/questionnairedevelopment/finalisingthe2011questionnaire/final-recommended-questions-2011-language_tcm77-183991.pdf

Box 1. Enhanced data collection for Public Health Specialty Recruitment, 2021 recruitment round.

Professional background was assumed based on the eligibility criteria applied by each candidate: whether medical or BOTM. Additionally, we reviewed the degree awarding institution for all applicants who applied through the medical route to sort this group into UK trained medical doctors and those who had trained outside the UK (termed International Medical Graduates, IMGs). Every year, a small number of people with a primary medical qualification apply through the BOTM eligibility criteria. Applicants who hold a primary medical qualification but do not hold UK medical registration and a licence to practise may apply through the BOTM route. It is therefore possible that some doctors, including IMGs, applied through this route, but their identity was not captured from our process, resulting in them being labelled as BOTM.

Data processing

Data processing and analysis was undertaken in STATA SE 17.0 for Mac. The primary outcome for the analyses was 'passing AC' defined as having achieved a minimum threshold score in each of the three

For peer review only - http://bmjopen.bmj.com/site/about/guidelines.xhtml

psychometric assessments. Sub-analyses were also undertaken (and are reported) for each psychometric test separately. Due to the comparatively small number of black and minority ethnic candidates, the ethnicity categories were aggregated upwards to create sufficiently large groups for analysis. Responses to the two new optional sociocultural questions (Box 1) were aggregated into: “Parental Degree” or “No parental degree”; and “English as main language” and “Not English as main language”.

Analytical approach

Descriptive analysis of passing the Assessment Centre and its constituent parts was undertaken with counts and percentages. Logistic regression was used for both univariable and multivariable analysis producing odds ratios (OR) and adjusted odds ratios (aOR) with 95% confidence intervals (CI), assuming two-tailed testing with an alpha of 0.05. Applicants for whom demographic data were missing are presented in descriptive analyses with count data but censored in regression analyses. Descriptive analyses where counts are <5 have been masked to reduce the risk of deductive disclosure and maintain privacy.

For peer review only

For peer review only - http://bmjopen.bmj.com/site/about/guidelines.xhtml

RESULTS (609 words)

There were 963 applicants for Public Health Specialty Recruitment in the 2021 round, of which 629 (65.3%) applicants passed eligibility checking and attended the Assessment Centre; the results presented are for these candidates only.

Of the candidates attending the Assessment Centre, approximately two-thirds were female (65.3%), just over half (57.7%) identified themselves as coming from white backgrounds (Table 1) and the median age was 32 years. In terms of professional background, 53.6% of AC attendees came from backgrounds other than medicine, while 11.6% and 34.8% were identified as coming from IMG and UK medical backgrounds respectively. The response rate to the enhanced question set (for those attending the Assessment Centre) was 96.0% in relation to parental qualification, but only 76.3% in relation to main language. Of those responding to these optional questions, 37.6% reported their parent or carer holding a degree-level qualification by the time they were 18 years old, and 93.5% reported their main language as being English.

Of those attending the Assessment Centre, 357 (56.8%) scored sufficiently well across the three assessments to ‘pass’ it.

For peer review only

For peer review only - http://bmjopen.bmj.com/site/about/guidelines.xhtml

Table 1. Descriptive analysis of demographic characteristics of candidates attending Assessment Centre 2021, with sub-analysis of Assessment Centre outcome (count and percentage).

	All candidates sitting AC n (%)	Candidates failing AC n (%)	Candidates passing AC n (%)
**Total [%]**	629	272 [43.2]	357 [56.8]
**Sex**			
- Male	199 (31.6)	89 (32.7)	110 (30.8)
- Female	411 (65.3)	177 (65.1)	234 (65.6)
- Other or not known	19 (3.0)	6 (2.2)	13 (3.6)
**Ethnic Group**			
- White British	304 (48.3)	98 (36.0)	206 (57.7)
- White Other	59 (9.4)	24 (8.8)	35 (9.8)
- Black	56 (8.9)	41 (15.1)	15 (4.2)
- Asian	114 (18.1)	65 (23.9)	49 (13.7)
- Mixed	32 (5.1)	12 (4.4)	20 (5.6)
- Chinese	11 (1.8)	7 (2.6)	4 (1.1)
- Other	15 (2.4)	8 (2.9)	7 (2.0)
- Not disclosed	38 (6.0)	17 (6.3)	21 (5.9)
**Age in years**			
- ≤29	209 (32.2)	61 (22.4)	148 (41.5)
- 30-34	181 (28.8)	77 (28.3)	104 (29.1)
- 35-39	96 (15.3)	53 (19.5)	43 (12.0)
- 40-44	70 (11.1)	42 (15.4)	28 (7.9)
- 45-49	33 (5.3)	21 (7.7)	12 (3.4)
- ≥50	9 (1.4)	5 (1.8)	4 (1.1)
- Not known	31 (4.9)	13 (4.8)	18 (5.0)
**Parent or carer highest qualification**			
- Degree	227 (36.1)	105 (38.6)	122 (34.2)
- No degree	377 (59.9)	159 (58.5)	218 (61.1)
- Not known	25 (4.0)	8 (2.9)	17 (4.8)
**Main language**			
- English	449 (71.4)	183 (67.3)	266 (74.5)
- Not English	31 (4.9)	18 (6.6)	13 (3.6)
- Not known	149 (23.7)	71 (26.1)	78 (21.9)
**Professional background**			
- BOTM	337 (53.6)	176 (64.7)	161 (45.1)
- IMG	73 (11.6)	57 (21.0)	16 (4.5)
- UK Medical	219 (34.8)	39 (14.3)	180 (50.4)

Breaking down the applicants by professional background (Table 2), female applicants outnumbered males across the groups but particularly so in the BOTM group (75.2% versus 25.0%). The median age was five years greater for BOTM candidates than among those from the UK medical graduate group. The BOTM group was more socioeconomically diverse than the two medical groupings with 41.6% (of responding applicants) reporting a parent or carer without a degree.

For peer review only - http://bmjopen.bmj.com/site/about/guidelines.xhtml

Table 2. Descriptive analysis of demographic characteristics of candidates by professional background in Assessment Centre 2021 (count and percentage).

	All candidates from backgrounds other than medicine n (%)	All international medical graduate candidates n (%)	All UK medical graduate candidates n (%)
**Total [%]**	337	73	219
**Sex**			
- Male	82 (24.8)	31 (43.1)	86 (41.3)
- Female	248 (75.2)	41 (56.9)	122 (58.7)
**Ethnic Group**			
- White British	191 (58.6)	<5	111 (55.0)
- White Other	30 (9.2)	13 (20.0)	16 (8.0)
- Black	24 (7.4)	23 (35.4)	9 (4.5)
- Asian	52 (16.0)	18 (27.7)	44 (22.0)
- Mixed	17 (5.2)	5 (7.7)	10 (5.0)
- Chinese	5 (1.5)	<5	5 (2.5)
- Other	7 (2.2)	<5	5 (2.5)
**Median age in years [interquartile range]**	34 [31-41]	31 [29-36]	29 [27-31]
**Parent or carer highest qualification**			
- Degree	192 (58.4)	50 (72.5)	135 (65.5)
- No degree	137 (41.6)	19 (27.5)	71 (34.5)
**Main language**			
- English	252 (95.8)	41 (71.9)	156 (97.5)
- Not English	11 (4.2)	16 (28.1)	<5

Logistic regression analyses (Table 3) suggested that that sex, age and main language were not statistically associated with passing the AC (termed hereafter 'progression'). However, there was statistically significant lower progression among applicants from black backgrounds (aOR 0.19, 95% CI 0.07 to 0.47) and Asian backgrounds (aOR 0.36, 95% CI 0.19-0.66). The association of lower progression probability with advancing age was not statistically significant after adjustment.

Professional background was also strongly associated with progression: compared to UK medical graduates, IMGs were more than 90% less likely to progress (aOR 0.07, 95% CI 0.03 to 0.17) and BOTM candidates approximately 80% less likely (aOR 0.21, 95% CI 0.11 to 0.39). Subgroup analysis of UK medical graduates only, where ethnicity was recorded (n = 200), showed a 14.2% percentage point gap between white British candidates (89.2% progressed) and those who were not white British (75.0%, p = 0.003).

For peer review only - http://bmjopen.bmj.com/site/about/guidelines.xhtml

Table 3. Univariable and multivariable logistic regression of demographic associations of candidates passing AC (odds ratios, adjusted odds ratios, 95% confidence intervals and p-values), with sub-group analysis of UK trained medical applicants (count, percentage and χ^2 p-value).

	**Odds ratio (95% CI)**	**p-value**	**Adjusted odds ratio* (95% CI)**	**p-value**
**Sex**				
- Male	Ref		Ref	
- Female	1.07 (0.76-1.50)	0.70	1.20 (0.73-1.97)	0.46
**Ethnic Group**				
- White British	Ref		Ref	
- White Other	0.69 (0.39 to 1.23)	0.21	1.10 (0.50 to 2.46)	0.89
- Black	0.17 (0.09 to 0.33)	<0.001	0.19 (0.07 to 0.47)	<0.001
- Asian	0.36 (0.23 to 0.56)	<0.001	0.36 (0.19 to 0.66)	<0.001
- Mixed	0.79 (0.37 to 1.69)	0.55	1.35 (0.49 to 3.69)	0.56
- Chinese	0.27 (0.08 to 0.95)	0.04	0.20 (0.03 to 1.58)	0.13
- Other	0.42 (0.15 to 1.18)	0.10	0.45 (0.11 to 1.80)	0.26
**Age in years****	0.92 (0.90 to 0.95)	<0.001	0.96 (0.93 to 1.00)	0.07
**Parent or carer highest qualification**				
- No degree	Ref		Ref	
- Degree	1.18 (0.85 to 1.64)	0.32	1.55 (0.99 to 2.43)	0.06
**Main language**				
- English	Ref		Ref	
- Not English	0.50 (0.34 to 1.04)	0.06	1.37 (0.52 to 3.67)	0.52
**Professional background**				
- BOTM	0.20 (0.13 to 0.30)	<0.001	0.21 (0.11 to 0.39)	<0.001
- IMG	0.06 (0.03 to 0.12)	<0.001	0.07 (0.03 to 0.17)	<0.001
- UK Medical	Ref		Ref	
Subgroup analysis of UK trained medical applicants only	*Passed AC* **n**	**(%)**	**χ^2 p-value**	
- White British (n = 111)	99	(89.2)	0.003	
- Ethnic minority (n = 89)	81	(75.0)		

* - adjustment includes sex, ethnic group, age, parent or carer degree status, first language and professional background.
** - age as a continuous variable in years.

For peer review only

Descriptive and inferential analysis of the RANRA, Watson-Glaser and SJT instruments separately demonstrated broadly similar results (Supplementary, Tables S1-3). The highest pass rate (79.3%) was achieved on the RANRA (numerical reasoning) assessment, followed by Watson-Glaser (74.1%) then SJT (73.9%).

Candidates from black backgrounds (black African, black Caribbean and black other) returned the lowest pass rates across all three instruments, followed by those from Asian backgrounds (Indian, Pakistani, Bangladeshi and other Asian). The performance gap by for those applicants from black backgrounds was

For peer review only - http://bmjopen.bmj.com/site/about/guidelines.xhtml

smallest on the SJT. The 'other' ethnic group also returned lower scores in univariable regression but after adjustment this was not the case. Younger age was associated with higher probability of passing – but the association was attenuated to a borderline finding following adjustment. UK medical graduates were significantly more likely to pass than the other two professional groups, with IMGs receiving the lowest scores overall. UK medical graduates showed the greatest performance gap with BOTM applicants in the RANRA (95.4% passing versus 74.5%) and the SJT (93.2% passing versus 67.4%).

Parental qualification was broadly not associated with performance (except weakly following adjustment in Watson-Glaser). Univariable regression suggested that main language other than English might be associated with lower scores across instruments; however, these associations were attenuated following adjustment in the multivariable regression.

For peer review only

For peer review only - http://bmjopen.bmj.com/site/about/guidelines.xhtml

DISCUSSION (1699 words)

Statement of principal findings

Coming from a black ethnic group, Asian ethnic group, or from a non-UK medical graduate background were all independently predictive of poorer performance across all three elements of the UK psychometric tests used in Public Health Specialty Recruitment in 2021. We detected no consistent pattern of lower-scoring performance associated with English as a second language, or from socioeconomic background (as measured by parental qualification). Increasing age may be associated with poorer performance, but some of the previously-noted performance gap appeared to be accounted for by confounding (possibly due to medical applicants, who perform better overall, tending to be younger). Yet even within the UK-trained medical cohort of applicants, there was differential attainment by ethnicity.[28]

The hypothesis, outlined in our previous paper,[21] that sociocultural factors (including first language) might be causing poorer performance in the SJT the highly time-pressured Watson-Glaser, was not supported by the analysis. We had also hypothesised that the use of SJTs in other medical application processes might have conferred an advantage to UK medical graduates; and language fluency has previously been associated with SJT performance in the recruitment process for general practice.[28] Yet the performance difference in the SJT was not substantially different to the gap observed in the RANRA. Instead, we observed consistent performance across all three instruments, with significantly lower performance by candidates from black and Asian backgrounds. Black candidates were 80% less likely than white British applicants to pass the Assessment Centre in 2021, and Asian candidates 60% less likely.

That these differences persisted even after adjustment for professional background and sub-categorisation of international medical graduates is deeply concerning. A further striking finding is that UK medical graduates were ten times more likely to pass the AC than international medical graduates. Once again, this difference persisted after multivariable adjustment.

Each of these three psychometric tests was developed and validated independently. Each test is designed to test a separate aspect of potential suitability for Public Health training. Yet similar patterns of differential

For peer review only - http://bmjopen.bmj.com/site/about/guidelines.xhtml

attainment are observed across all three. Together, these findings add to the literature that suggests differential attainment is present across a wide range of psychometric tests.[29,3029]

The earlier working hypothesis that age was likely confounded by professional background appears broadly justified although there remains some residual association after adjustment. The wider distribution of ages in the BOTM group may explain some of this difference. Although the two medical groups were socioeconomically more advantaged overall than the BOTM group, it was notable that the socioeconomic metric was not strongly associated with progression. However, the cohort entering the process appears more homogeneously drawn from higher socioeconomic groups than the UK population average. Accordingly, a type II error cannot be ruled out.

Strengths and weaknesses of the study

To our knowledge, this is the first study to systematically analyse differential attainment in a UK national selection process: while other analyses have made high-level comparisons, this study has examined in detail the intricacies of a single large-scale selection process and sought to anticipate confounding through the use of bespoke sociocultural questions, prospectively integrated into the process. Yet residual confounding cannot be ruled-out: the sociocultural variables used in the regression are imperfect, and it is very possible that other confounding variables were in-play. Our findings have important implications for both Public Health as a specialty in the UK, and for the many other medical specialties that employ psychometric testing in their selection processes.

Among the study's weaknesses is that ethnic groupings had to be aggregated to mitigate the analytical risk arising from small numbers. Accordingly, Type I and Type II errors cannot be ruled out. However, the consistency and strength of the findings around ethnicity and professional background suggest that a Type I error is highly unlikely in this context. The single-year nature of the dataset is a limitation, but for those variables measured since 2018[21] the associations are consistent over four years of recruitment: we note the proportion of candidates passing the AC in this year (56.8%) is very similar to the preceding year's cohort (56.5%). It is not possible to infer what effect the COVID-19 pandemic might have had (with the Assessment Centre taking place in February 2021 and most candidates completing the tests at home rather than in a commercial test centre.

For peer review only - http://bmjopen.bmj.com/site/about/guidelines.xhtml
For peer review only

The application of the sociocultural questions, while drawn from best available evidence on the measurement of socioeconomic status, may have been insufficient to detect differences, and the concept of socioeconomic status is complex. We note that “first language” may not be easy to define for those who have grown up bilingual. The response rate on parental qualification (at 96.0%) was very high; for the language question response rate was lower at 76.3%. The reason for the differential response rate on two sequential questions is unclear.

Strengths and weaknesses in other studies, discussing important differences in results

As already noted, in the context of the published literature, these differential attainment gaps are not as surprising as they might at first appear.[2] There is already extensive evidence of differential attainment relating to ethnicity across medical training in the UK.[31] Likewise, the broader evidence base on psychometric testing shows relatively consistent findings that older and ethnic minority candidates generally perform less well on standardised testing.[29] While there remains some uncertainty over the precise drivers, possible explanations include specific barriers, such as differential access to networks, familiarity with psychometric testing,[32] test taker perception,[29] stereotype threat,[33] through to systemic disadvantage and structural racism occurring through the life-course. Such putative explanatory variables are extremely difficult to measure or indeed analyse in a quantitative methodology.

Meaning of the study: possible explanations and implications for clinicians and policymakers

Due to Public Health recruitment drawing on a more diverse candidate pool than most other medical specialties (that require a primary medical qualification), comparing Public Health recruitment with other national programmes is difficult. Successful completion of medical school and foundation training is likely to create a more homogeneous and high-performing cohort of applicants. However, this study suggests that the observed differential attainment cannot be attributed to confounding alone: likely differential attainment was observed even within the UK medical graduate group. It appears that the system is unfairly discriminating against certain candidates.

For peer review only

For peer review only - http://bmjopen.bmj.com/site/about/guidelines.xhtml

While the status quo is unacceptable, we recognise the validity of the current recruitment process which has demonstrated that the candidates selected in the current process progress well in later Public Health training.[17] However there is some potential circularity to such a validation: that by selecting out people (through differential attainment) before training scheme entry, the trainees who do proceed are less subject to differential attainment later, leading to the appearance of a valid process. Differential attainment has been seen in postgraduate training in other specialties.[11] Any policy responses to this analysis need to preserve the strengths of the current system. Reducing a cohort of applicants from approximately 1000 to 100 needs a scalable, objective and practicable approach, and there is little doubt that the current system achieves some, if not most, of those objectives, at least in part.

In our preceding paper,[21] we outlined some of the potential responses to apparent differential attainment in the recruitment process more generally. In the context of these findings from the Assessment Centre stage specifically, the potential response options include a comprehensive review of the psychometric tests used to devise an assessment process and additional support for candidates from disadvantaged groups.

The implications of these findings reach well beyond Public Health. While most national recruitment processes draw only on medical graduates, the apparent effect of ethnicity among UK medical graduates, coupled with the apparent gap among international medical graduates, has important ramifications for all medical selection processes that are using psychometric tests. We propose that the assumption for selector teams must now be that there is differential attainment in all psychometric test instruments until proven otherwise. We encourage other specialties to explore the data on their own processes and to integrate prospective monitoring processes such as the sociocultural questions that we have employed in Public Health. Such efforts are imperative if we are to mitigate the risk of group-think perpetuating the selection of people who think like the 'establishment'.

Unanswered questions and future research

This study has identified likely differential attainment in the current national selection process for Public Health. And while it increasingly appears that differential attainment is a feature of psychometric testing in its present form, we are unable to clearly recommend mitigation that would be both simple and uncontentious.

For peer review only - http://bmjopen.bmj.com/site/about/guidelines.xhtml

While there is a need for additional research, as a medical profession we must be careful to avoid additional analysis becoming a rationale for delaying our response.

A range of additional variables may be useful. Neurodiversity was not included in the analysis although candidates declaring such conditions were provided with reasonable adjustments which included additional time and / or adjustments to the exam logistics. Previous work among trainees in general practice has observed higher prevalence of dyslexia among international medical graduates who had failed their first applied knowledge test (AKT).[34] Likewise, prior education or educational performance was also not included in the analysis. Previous has work has suggested possible intersectional associations with ethnicity in respect of prior education.[35] Capturing such data (while acknowledging the arguably wider range of educational backgrounds from which Public Health applicants come) is an area for potential further work.

There is also a more fundamental limitation of quantitative approaches to 'measuring' diversity:[36] the quantitative paradigm is problematic in recognising intersectionality as well as other contributing factors that motivate fairer selection. Some psychometricians have also challenged the assumption (the so-called Cultural Test Bias Hypothesis) that the observation of mean differences can be inferred as bias.[37] But in the absence of alternatives and the sheer volume of applicants and the differences observed, this analysis is an important start.

Conclusions

All doctors and Public Health professionals need to have confidence in national systems of selection. We need a senior medical and public health workforce that better reflects the populations from which it is drawn and which they seek to serve. The drivers of differential attainment are complex, and policy responses even more so.[2,36] However, Public Health is a specialty that values diversity and promotes equity of opportunity. It is vital that we create a fairer future for doctors, health care and policy professionals.

For peer review only - http://bmjopen.bmj.com/site/about/guidelines.xhtml

Funding

Imperial College London is grateful for support from the NW London NIHR Applied Research Collaboration. The views expressed in this publication are those of the authors and not necessarily those of the NIHR or the Department of Health and Social Care.

Statement of involvement and engagement

Neither patients nor public were involved in the design, conduct, reporting or dissemination of this work.

Data sharing statement

The dataset was provided to the research team by Health Education England East Midlands for the analysis. Requests to access and analyse this dataset should be made to Health Education England East Midlands (https://www.eastmidlandsdeanery.nhs.uk/contact_us).

Competing interests

RJP is a Fellow of the Faculty of Public Health and former Technical Assessment Lead within the national Public Health Recruitment Executive Group hosted by Health Education England. FB is a Member of the Faculty of Public Health and a member of the Faculty's Special Interest Group in Equality and Diversity. FB is also a Specialty Registrar in Public Health. AM is a Fellow of the Faculty of Public Health. MR is a Fellow of the Faculty of Public Health and former Medical Advisor to NHS England on Workforce Race Equality.

Contributorship statement

RJP and FB conceived the study and undertook the analyses that are presented in this paper. RJP drafted the original version of this manuscript and amendments following peer review. FB, GS, AM and MR reviewed and revised the draft. MR is the guarantor of this paper.

Ethics approval statement

The analyses are evaluation and the data were taken from an administrative dataset. Permission was granted by the data owner to use these data for the explicit analytical purposes of this paper, but these analyses and approach were not anticipated at the time that the data were collected.

For peer review only

For peer review only - http://bmjopen.bmj.com/site/about/guidelines.xhtml

References

1. Nagpaul, C. Racism is wrecking the lives of doctors, affecting patient care, and jeopardising services. *BMJ* **377**, o1515 (2022).
2. Woolf, K. Differential attainment in medical education and training. *BMJ* **368**, (2020).
3. Patterson, F., Tiffin, P. A., Lopes, S. & Zibarras, L. Unpacking the dark variance of differential attainment on examinations in overseas graduates. *Med Educ* **52**, 736–746 (2018).
4. Tiffin, P. A. & Paton, L. W. Differential attainment in the MRCPsych according to ethnicity and place of qualification between 2013 and 2018: a UK cohort study. *Postgrad Med J* **97**, 764–776 (2021).
5. Kar, P. Partha Kar: Racism in the medical workforce—five initial steps. *BMJ* **377**, o1280 (2022).
6. Gravlee, C. C. How race becomes biology: embodiment of social inequality. *Am J Phys Anthropol* **139**, 47–57 (2009).
7. General Medical Council. What is differential attainment? https://www.gmc-uk.org/education/standards-guidance-and-curricula/projects/differential-attainment/what-is-differential-attainment (2022).
8. McManus, I. C., Dewberry, C., Nicholson, S. & Dowell, J. S. The UKCAT-12 study: Educational attainment, aptitude test performance, demographic and socio-economic contextual factors as predictors of first year outcome in a cross-sectional collaborative study of 12 UK medical schools. *BMC Med* **11**, (2013).
9. Davis, D. *et al.* Do racial and ethnic group differences in performance on the MCAT exam reflect test bias? *Academic Medicine* **88**, 593–602 (2013).
10. Hope, D. *et al.* Measuring differential attainment: a longitudinal analysis of assessment results for 1512 medical students at four Scottish medical schools. *BMJ Open* **11**, (2021).
11. Shah, R. & Ahluwalia, S. The challenges of understanding differential attainment in postgraduate medical education. *British Journal of General Practice* **69**, 426–427 (2019).
12. Royal College of Physicians, L. *2017 survey of medical certificate of completion of training (CCT) holders' career progression*. https://www.rcplondon.ac.uk/projects/outputs/2017-survey-medical-certificate-completion-training-cct-holders-career-progression (2018).
13. Powell, R. A. *et al.* Tackling racism in UK health research. *BMJ* **376**, (2022).
14. Consul, N., Strax, R., DeBenedectis, C. M. & Kagetsu, N. J. Mitigating Unconscious Bias in Recruitment and Hiring. *J Am Coll Radiol* **18**, 769–773 (2021).

For peer review only

For peer review only - http://bmjopen.bmj.com/site/about/guidelines.xhtml

15. Manchester tackles failure rate of Asian students. *BMJ* **310**, 209 (1995).

16. Koczwara, A. *et al.* Evaluating cognitive ability, knowledge tests and situational judgement tests for postgraduate selection. *Med Educ* **46**, 399–408 (2012).

17. Pashayan, N. *et al.* Evaluation of recruitment and selection for specialty training in public health: interim results of a prospective cohort study to measure the predictive validity of the selection process. *J Public Health (Bangkok)* **38**, e194–e200 (2016).

18. Work Psychology Group. *Multi-Specialty Recruitment Assessment (MSRA) - Test Blueprint & Information*. https://heeoe.hee.nhs.uk/sites/default/files/msra_test_blueprint_information_nov_2019.pdf (2019).

19. Lievens, F. & Patterson, F. The Validity and Incremental Validity of Knowledge Tests, Low-Fidelity Simulations, and High-Fidelity Simulations for Predicting Job Performance in Advanced-Level High-Stakes Selection. *Journal of Applied Psychology* **96**, 927–940 (2011).

20. Tekkis, N. P. *et al.* The impact of the COVID-19 pandemic on UK medical education. A nationwide student survey. *Med Teach* (2021) doi:10.1080/0142159X.2021.1962835/SUPPL_FILE/IMTE_A_1962835_SM5137.DOCX.

21. Bury, F., Rao, M. & Pinder, R. Differential attainment in Public Health specialty training recruitment in the United Kingdom: an observational analysis of applicants from 2018 to 2020. *J Public Health* (2022).

22. Rust, J. *Advanced Numerical Reasoning Appraisal TM (ANRA) Manual*. https://docplayer.net/44427444-Rust-advanced-numerical-reasoning-appraisal.html (2006).

23. Watson, G. & Glaser, E. M. *Watson-Glaser TM II Critical Thinking Appraisal Technical Manual and User's Guide*. https://www.yumpu.com/en/document/read/41941997/watson-glaser-user-guide-and-technical-manual-talentlens (2010).

24. Iacobucci, G. Specialty training: ethnic minority doctors' reduced chance of being appointed is "unacceptable". *BMJ* **368**, m479 (2020).

25. Information Commissioner's Office. Principle (c): Data minimisation. https://ico.org.uk/for-organisations/guide-to-data-protection/guide-to-the-general-data-protection-regulation-gdpr/principles/data-minimisation/ (2022).

26. *Annex A - Evaluation of measures of socio-economic background*. (2018).

For peer review only - http://bmjopen.bmj.com/site/about/guidelines.xhtml

27. Census 2011. *Final recommended questions for the 2011 Census in England and Wales, Language*. (2009).

28. Patterson, F., Tiffin, P. A., Lopes, S. & Zibarras, L. Unpacking the dark variance of differential attainment on examinations in overseas graduates. *Med Educ* **52**, 736–746 (2018).

29. Hough, L. M., Oswald, F. L. & Ployhart, R. E. Determinants, Detection and Amelioration of Adverse Impact in Personnel Selection Procedures: Issues, Evidence and Lessons Learned. *International Journal of Selection and Assessment* **9**, 152–194 (2001).

30. Sackett, P. R., Zhang, C., Berry, C. M. & Lievens, F. Revisiting Meta-Analytic Estimates of Validity in Personnel Selection: Addressing Systematic Overcorrection for Restriction of Range. *Journal of Applied Psychology* (2021) doi:10.1037/APL0000994.

31. Linton, S. Taking the difference out of attainment. *BMJ* **368**, (2020).

32. Hinton, D. P. Uncovering the root causes of ethnic differences in ability testing: Differential test functioning, test familiarity and trait optimism as explanations of ethnic group differences. (Aston University, 2014).

33. Steele, C. M. A threat in the air. How stereotypes shape intellectual identity and performance. *Am Psychol* **52**, 613–629 (1997).

34. Asghar, Z. B. *et al.* Performance of candidates disclosing dyslexia with other candidates in a UK medical licensing examination: cross-sectional study. *Postgrad Med J* **94**, 198–203 (2018).

35. McManus, I. C., Woolf, K., Dacre, J., Paice, E. & Dewberry, C. The Academic Backbone: Longitudinal continuities in educational achievement from secondary school and medical school to MRCP(UK) and the specialist register in UK medical students and doctors. *BMC Med* **11**, 1–27 (2013).

36. Clapp, J. T. & Gordon, E. K. Selecting trainees: Too much focus on predictive metrics, not enough on holistic review. *Med Educ* **56**, 139–141 (2022).

37. Reynolds, C. R., Altmann, R. A. & Allen, D. N. The Problem of Bias in Psychological Assessment. *Mastering Modern Psychological Testing* 573–613 (2021) doi:10.1007/978-3-030-59455-8_15.

For peer review only

For peer review only - http://bmjopen.bmj.com/site/about/guidelines.xhtml

Differential attainment in specialty training recruitment in the United Kingdom: an observational analysis of the impact of psychometric testing assessment in Public Health postgraduate selection.

Submission for BMJ OPEN

STRICTLY CONFIDENTIAL

Authors

Richard J Pinder[1] **Corresponding author; richard.pinder@imperial.ac.uk**

ORCID: 0000-0002-7010-6009;

Fran Bury[1] ORCID: 0000-0002-6305-1994

Ganesh Sathyamoorthy[1,2, 3]

Azeem Majeed[1,3] ORCID: 0000-0002-2357-9858

Mala Rao[1,2,3] ORCID: 0000-0001-5504-6303

Affiliations

1. Department of Primary Care and Public Health, School of Public Health, Imperial College London
2. Ethnicity and Health Unit, Department of Primary Care and Public Health, Imperial College London
3. NIHR Applied Research Collaboration Northwest London

Key words

Public health, recruitment, medical training, ethnicity, differential attainment

SUPPLEMENTARY MATERIALS

For peer review only - http://bmjopen.bmj.com/site/about/guidelines.xhtml

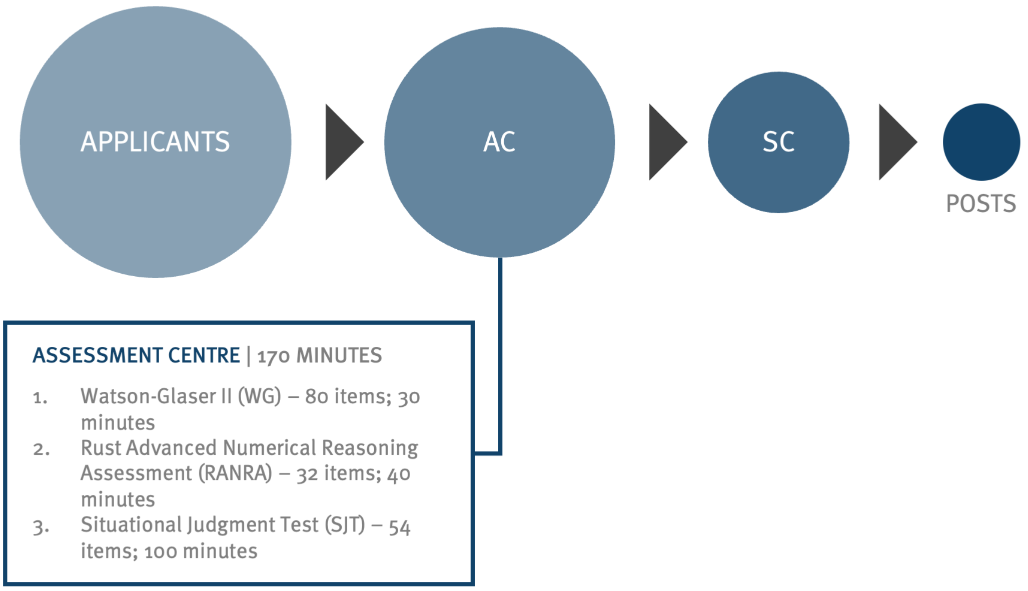

Supplementary Figure 1. Recruitment cycle diagram for Public Health specialty training, 2009-2020

For peer review only

For peer review only - http://bmjopen.bmj.com/site/about/guidelines.xhtml

Supplementary Table 1. Univariable and multivariable logistic regression of demographic associations of candidates passing RANRA (count, percentage, odds ratios, adjusted odds ratios, 95% confidence intervals and p-values).

	Passed RANRA (% of those sitting)	Odds ratio (95% CI)	p-value	Adjusted odds ratio* (95% CI)	p-value
**Overall**	499 (79.3)				
**Sex**					
- Male	157 (78.9)	Ref		Ref	
- Female	324 (78.8)	1.00 (0.66 to 1.51)	0.99	1.06 (0.59 to 1.90)	0.85
**Ethnic Group**					
- White British	270 (88.8)	Ref		Ref	
- White Other	47 (79.7)	0.49 (0.24 to 1.02)	0.06	0.90 (0.33 to 2.48)	0.85
- Black	31 (55.4)	0.16 (0.08 to 0.30)	<0.001	0.19 (0.08 to 0.44)	<0.001
- Asian	79 (69.3)	0.28 (0.17 to 0.49)	<0.001	0.35 (0.16 to 0.71)	<0.001
- Mixed	25 (78.1)	0.45 (0.18 to 1.11)	0.09	0.63 (0.20 to 1.99)	0.43
- Chinese	8 (72.7)	0.34 (0.08 to 1.33)	0.12	0.55 (0.05 to 6.21)	0.63
- Other	10 (66.7)	0.25 (0.08 to 0.78)	0.02	0.31 (0.07 to 1.36)	0.12
**Age in years****	Range: 24 to 55	0.92 (0.89 to 0.95)	<0.001	0.95 (0.91 to 0.99)	0.02
**Parent or carer highest qualification**					
- No degree	180 (79.3)	Ref		Ref	
- Degree	300 (79.6)	1.02 (0.68 to 1.53)	0.93	1.16 (0.67 to 1.77)	0.60
**Main language**					
- English	363 (80.9)	Ref		Ref	
- Not English	19 (61.3)	0.38 (0.18 to 0.80)	0.01	0.66 (0.25 to 1.77)	0.41
**Professional background**					
- BOTM	251 (74.5)	0.14 (0.07 to 0.28)	<0.001	0.18 (0.07 to 0.47)	<0.001
- IMG	39 (53.4)	0.05 (0.03 to 0.12)	<0.001	0.10 (0.04 to 0.32)	<0.001
- UK Medical	209 (95.4)	Ref		Ref	

* - adjustment includes sex, ethnic group, age, parent or carer degree status, main language and professional background.
** - age as a continuous variable in years.

For peer review only - http://bmjopen.bmj.com/site/about/guidelines.xhtml

Supplementary Table 2. Univariable and multivariable logistic regression of demographic associations of candidates passing Watson-Glaser (count, percentage, odds ratios, adjusted odds ratios, 95% confidence intervals and p-values).

	Passed WG (% of those sitting)	Odds ratio (95% CI)	p-value	Adjusted odds ratio* (95% CI)	p-value
**Overall**	466 (74.1)				
**Sex**					
- Male	140 (70.4)	Ref			
- Female	310 (75.4)	1.29 (0.89-1.89)	0.18	1.25 (0.73-2.17)	0.41
**Ethnic Group**					
- White British	255 (83.9)	Ref		Ref	
- White Other	47 (79.7)	0.75 (0.37-1.52)	0.43	1.22 (0.45-3.30)	0.70
- Black	26 (46.4)	0.17 (0.91-0.31)	<0.001	0.20 (0.09-0.46)	<0.001
- Asian	72 (63.2)	0.33 (0.20-0.54)	<0.001	0.30 (0.16-0.58)	<0.001
- Mixed	26 (81.3)	0.83 (0.33-2.13)	0.70	1.24 (0.36-4.30)	0.73
- Chinese	6 (54.6)	0.23 (0.68-0.79)	0.02	0.22 (0.03-1.74)	0.15
- Other	8 (53.3)	0.22 (0.08-0.63)	0.01	0.25 (0.06-1.00)	0.05
**Age in years****	Range: 24-54	0.94 (0.92-0.97)	<0.001	0.95 (0.92-1.00)	0.03
**Parent or carer highest qualification**					
- No degree	159 (70.0)	Ref		Ref	
- Degree	287 (76.1)	1.36 (0.94-1.97)	0.10	1.78 (1.07-2.98)	0.03
**Main language**					
- English	347 (77.3)	Ref		Ref	
- Not English	19 (61.3)	0.47 (0.22-0.99)	0.05	0.84 (0.31-2.23)	0.72
**Professional background**					
- BOTM	243 (72.1)	0.35 (0.22-0.56)	<0.001	0.39 (0.19-0.80)	0.01
- IMG	30 (41.1)	0.09 (0.05-0.17)	<0.001	0.13 (0.05-0.33)	<0.001
- UK Medical	193 (88.1)	Ref		Ref	

* - adjustment includes sex, ethnic group, age, parent or carer degree status, main language and professional background.
** - age as a continuous variable in years.

For peer review only - http://bmjopen.bmj.com/site/about/guidelines.xhtml

Supplementary Table 3. Univariable and multivariable logistic regression of demographic associations of candidates passing Situational Judgment Test (count, percentage, odds ratios, adjusted odds ratios, 95% confidence intervals and p-values).

	Passed SJT (% of those sitting)	Odds ratio (95% CI)	p-value	Adjusted odds ratio* (95% CI)	p-value
**Overall**	465 (73.9)				
**Sex**					
- Male	143 (71.9)	Ref		Ref	
- Female	307 (74.7)	1.16 (0.79-1.69)	0.46	1.59 (0.94-2.69)	0.09
**Ethnic Group**					
- White British	251 (82.6)	Ref		Ref	
- White Other	44 (74.6)	0.62 (0.32-1.19)	0.15	1.25 (0.49-3.21)	0.64
- Black	30 (53.6)	0.24 (0.13-0.45)	<0.001	0.39 (0.17-0.88)	0.02
- Asian	73 (64.0)	0.38 (0.23-0.61)	<0.001	0.38 (0.20-0.74)	0.01
- Mixed	22 (68.8)	0.46 (0.21-1.04)	0.06	0.74 (0.26-2.11)	0.57
- Chinese	7 (63.6)	0.37 (0.10-1.31)	0.12	0.26 (0.03-2.24)	0.22
- Other	9 (60.0)	0.32 (0.11-0.93)	0.04	0.30 (0.07-1.22)	0.09
**Age in years****	Range: 24-50	0.93 (0.90-0.95)		0.97 (0.93-1.01)	0.11
**Parent or carer highest qualification**					
- No degree	164 (72.3)	Ref		Ref	
- Degree	280 (74.3)	1.11 (0.77-1.61)	0.59	1.36 (0.83-2.23)	0.22
**First language**					
- English	339 (75.5)	Ref		Ref	
- Other	17 (54.8)	0.39 (0.19-0.83)	0.01	1.02 (0.68-2.71)	0.97
**Professional background**					
- BOTM	227 (67.4)	0.15 (0.09-0.27)	<0.001	0.16 (0.07-0.35)	<0.001
- IMG	34 (46.6)	0.06 (0.03-0.13)	<0.001	0.07 (0.03-0.19)	<0.001
- UK Medical	204 (93.2)	Ref		Ref	

* - adjustment includes sex, ethnic group, age, parent or carer degree status, main language and professional background.
** - age as a continuous variable in years.

For peer review only - http://bmjopen.bmj.com/site/about/guidelines.xhtml

STROBE Statement—checklist of items that should be included in reports of observational studies

	Item No	Recommendation	Page No
**Title and abstract**	1	(*a*) Indicate the study's design with a commonly used term in the title or the abstract	Yes, Page 1. "…an observational analysis…"
		(*b*) Provide in the abstract an informative and balanced summary of what was done and what was found	Yes, Page 2.
**Introduction**			
Background/rationale	2	Explain the scientific background and rationale for the investigation being reported	Yes, Page 4-5.
Objectives	3	State specific objectives, including any prespecified hypotheses	Yes, final two sentences; Page 5.
**Methods**			
Study design	4	Present key elements of study design early in the paper	Yes, paragraph 2, Page 8
Setting	5	Describe the setting, locations, and relevant dates, including periods of recruitment, exposure, follow-up, and data collection	Yes, Page 6 (including footnote [a]).
Participants	6	(*a*) *Cohort study*—Give the eligibility criteria, and the sources and methods of selection of participants. Describe methods of follow-up *Case-control study*—Give the eligibility criteria, and the sources and methods of case ascertainment and control selection. Give the rationale for the choice of cases and controls *Cross-sectional study*—Give the eligibility criteria, and the sources and methods of selection of participants	Observational study, technically prospective cohort by design. This criterion is not immediately relevant to this evaluation-driven approach. However eligibility criteria, sources and follow-up are described in the methods section.
		(*b*) *Cohort study*—For matched studies, give matching criteria and number of exposed and unexposed *Case-control study*—For matched studies, give matching criteria and the number of controls per case	N/A
Variables	7	Clearly define all outcomes, exposures, predictors, potential confounders, and effect modifiers. Give diagnostic criteria, if applicable	Yes, Box 1 page 7. All elements described pages 7-8.
Data sources/ measurement	8*	For each variable of interest, give sources of data and details of methods of assessment (measurement). Describe	Yes. First paragraph, Page 6/

For peer review only

		comparability of assessment methods if there is more than one group	
Bias	9	Describe any efforts to address potential sources of bias	Yes. Regression approach outlined on page 8.
Study size	10	Explain how the study size was arrived at	Yes. First paragraph, page 9.
Quantitative variables	11	Explain how quantitative variables were handled in the analyses. If applicable, describe which groupings were chosen and why	Yes. Table 1, page 10.
Statistical methods	12	(*a*) Describe all statistical methods, including those used to control for confounding	Yes. Page 8.
		(*b*) Describe any methods used to examine subgroups and interactions	Yes. Table 2 legend explains the breakdown by professional grouping. Supplementary tables provide more granular analysis by psychometric instrument.
		(*c*) Explain how missing data were addressed	Yes. Page 8, paragraph 2.
		(*d*) *Cohort study*—If applicable, explain how loss to follow-up was addressed<br>*Case-control study*—If applicable, explain how matching of cases and controls was addressed<br>*Cross-sectional study*—If applicable, describe analytical methods taking account of sampling strategy	There was no loss to follow up as this was an observational analysis /evaluation with a complete data set.
		(*e*) Describe any sensitivity analyses	Provided through supplementary tables S1-S3.

Continued on next page

For peer review only

**Results**			
Participants	13*	(a) Report numbers of individuals at each stage of study—eg numbers potentially eligible, examined for eligibility, confirmed eligible, included in the study, completing follow-up, and analysed	Yes. Paragraph 1, Page 9.
		(b) Give reasons for non-participation at each stage	Yes. Not applicable as selection process and this is an analysis of a single stage.
		(c) Consider use of a flow diagram	This was not felt necessary, but can be added at the request of the editor.
Descriptive data	14*	(a) Give characteristics of study participants (eg demographic, clinical, social) and information on exposures and potential confounders	Yes. Table 1 (Page 10) provides breakdown, and Table 2 provides additional data for confounding by professional background / route of application.
		(b) Indicate number of participants with missing data for each variable of interest	Yes; "Not known" is provided as a line for each variable in Table 1.
		(c) *Cohort study*—Summarise follow-up time (eg, average and total amount)	N/A
Outcome data	15*	*Cohort study*—Report numbers of outcome events or summary measures over time	Provided in final column of Table 1.
		Case-control study—Report numbers in each exposure category, or summary measures of exposure	N/A
		Cross-sectional study—Report numbers of outcome events or summary measures	N/A
Main results	16	(*a*) Give unadjusted estimates and, if applicable, confounder-adjusted estimates and their precision (eg, 95% confidence interval). Make clear which confounders were adjusted for and why they were included	Yes. Table 3 – point estimate odds ratio with 95% confidence intervals – for both univariable and multivariable analyses.
		(*b*) Report category boundaries when continuous variables were categorized	N/A
		(*c*) If relevant, consider translating estimates of relative risk into absolute risk for a meaningful time period	N/A
Other analyses	17	Report other analyses done—eg analyses of subgroups and interactions, and sensitivity analyses	Yes. Supplementary tables S1-S3 provide more granular analysis.
**Discussion**			
Key results	18	Summarise key results with reference to study objectives	Yes – Paragraph 1, page 14.
Limitations	19	Discuss limitations of the study, taking into account sources of potential bias or imprecision. Discuss both direction and magnitude of any potential bias	Yes, Paragraphs 2 and 3, page 15.
Interpretation	20	Give a cautious overall interpretation of results considering objectives, limitations, multiplicity of analyses, results from similar studies, and other relevant evidence	Yes, throughout Discussion.

For peer review only

For peer review only - http://bmjopen.bmj.com/site/about/guidelines.xhtml

Generalisability	21	Discuss the generalisability (external validity) of the study results	Yes. Paragraph 1, page 17.
**Other information**			
Funding	22	Give the source of funding and the role of the funders for the present study and, if applicable, for the original study on which the present article is based	Yes – in acknowledgements, page 18.

*Give information separately for cases and controls in case-control studies and, if applicable, for exposed and unexposed groups in cohort and cross-sectional studies.

Note: An Explanation and Elaboration article discusses each checklist item and gives methodological background and published examples of transparent reporting. The STROBE checklist is best used in conjunction with this article (freely available on the Web sites of PLoS Medicine at http://www.plosmedicine.org/, Annals of Internal Medicine at http://www.annals.org/, and Epidemiology at http://www.epidem.com/). Information on the STROBE Initiative is available at www.strobe-statement.org.

For peer review only
